# A Neural Circuit For Bergamot Essential Oil‐Induced Anxiolytic Effects

**DOI:** 10.1002/advs.202406766

**Published:** 2024-11-02

**Authors:** Meng‐Yu Zhu, Wan‐Ying Dong, Jin‐Rong Guo, Ji‐Ye Huang, Ping‐Kai Cheng, Yumeng Yang, An Liu, Xin‐Lu Yang, Xia Zhu, Zhi Zhang, Yuanyin Wang, Wenjuan Tao

**Affiliations:** ^1^ College & Hospital of Stomatology Anhui Medical University Key Lab of Oral Diseases Research of Anhui Province Hefei 230032 China; ^2^ Department of Physiology Anhui Provincial Key Laboratory for Brain Bank Construction and Resource Utilization School of Basic Medical Sciences Anhui Medical University Hefei 230032 China; ^3^ Department of Anesthesiology The First Affiliated Hospital of USTC Division of Life Sciences and Medicine University of Science and Technology of China Hefei 230026 China; ^4^ Center for Advanced Interdisciplinary Science and Biomedicine Institute of Health and Medicine Division of Life Sciences and Medicine University of Science and Technology of China Hefei 230026 China

**Keywords:** anxiolytic effects, bergamot essential oil, chemogenetic manipulations, neural circuits, optogenetic manipulations

## Abstract

Aromatic essential oils have been shown to relieve anxiety and enhance relaxation, although the neural circuits underlying these effects have remained unknown. Here, it is found that treatment with 1.0% bergamot essential oil (BEO) exerts anxiolytic‐like effects through a neural circuit projecting from the anterior olfactory nucleus (AON) to the anterior cingulate cortex (ACC) in acute restraint stress model mice. Collectively, in vivo two‐photon calcium imaging, viral tracing, and whole‐cell patch clamp recordings show that inhalation exposure to 1.0% BEO can activate glutamatergic projections from the AON to GABAergic neurons in the ACC, which drives inhibition of local glutamatergic neurons (AON^Glu^→ACC^GABA→Glu^). Optogenetic or chemogenetic manipulation of this pathway can recapitulate or abolish the BEO‐induced anxiolytic‐like behavioral effects in mice with ARS. Beyond depicting a previously unrecognized pathway involved in stress response, this study provides a circuit mechanism for the effects of BEO and suggests a potential target for anxiety treatment.

## Graphical abstract

Using state‐of‐the‐art neuroscience tools and methods, this study shows that glutamatergic neurons in the anterior olfactory nucleus project to the anterior cingulate cortex to mediate the anxiolytic effects of bergamot essential oil inhalation in mice.

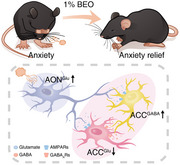

## 1 Introduction

Pharmacological interventions are widely used to reduce anxiety but are often accompanied by many potential adverse effects, such as confusion, fatigue, and addiction among others.^[^ ^1^ ^]^ Therefore, safe and evidence‐based complementary therapies may offer significant benefits in the management of anxiety. Aromatherapy with essential oils is rapidly gaining popularity as a complementary therapy worldwide. Aromatherapy (that is, inhalation exposure) with bergamot essential oil (BEO), in particular, has been shown to alleviate pain‐ and anxiety‐related behaviors in mice in several studies.^[^ ^2^ ^]^ Interestingly, these findings uncover a link between emotion and olfactory perception at the behavioral level. The mechanisms underlying the therapeutic efficacy of essential oils in alleviating anxiety remain unclear.

Olfactory perception is initiated by the binding of scent molecules with cognate receptors and terminates in the higher cerebral cortex.^[^ ^3^ ^]^ Olfactory stimulation may improve mood disorders. Regular exposure to odors with “‘smell training”’ fosters the processing of olfactory information.^[^ ^4^ ^]^ In this sensory pathway, the anterior olfactory nucleus (AON) is the first brain region to receive afferent inputs from the olfactory bulb (OB).^[^ ^5^ ^]^ In the complex neural network involved in the sense of smell, the AON has been shown to perform essential functions in the recognition and interpretation of various odors and plays a prominent role in the state‐dependent processing of olfactory behaviors.^[^ ^6^ ^]^ Electroencephalogram (EEG) data have shown that the neural representation of pleasant or unpleasant odor information spreads rapidly from the olfactory areas to regions associated with emotional processing, including the orbitofrontal cortex, anterior cingulate cortex (ACC), and bilateral insular cortex.^[^ ^7^ ^]^ However, the precise cell‐type specific organization and the function of the neural circuit for olfactory‐regulated anxiety remain unknown.

To investigate how the inhalation of essential oils could potentially relieve anxiety via activation of olfactory processing regions, we first found that BEO inhalation could reduce anxiety‐like behaviors in acute restraint stress (ARS) mice. We then identified the AON activated in response to BEO inhalation, and found that chemogenetic inhibition of the AON resulted in abolishing these anxiolytic‐like effects of BEO. We then defined the neural circuits downstream of the AON and focused on the ACC, in particular, based on both the preponderance of AON projections to this limbic region and its well‐established role in anxiety disorders. Dissection of the functional organization of this BEO‐responsive circuit revealed that glutamatergic neurons of the anterior olfactory nucleus project to local GABAergic neurons in ACC, which in turn innervate ACC glutamatergic neurons, resulting in an ACC^GABA→Glu^ microcircuit that can inhibit stress‐induced hyperexcitation of ACC^Glu^ neurons and ultimately alleviate anxiety‐like behaviors in mice. Our results thus define the circuit mechanisms underlying the anxiolytic‐like effects of BEO inhalation in mice with acute stress.

## 2 Results

### 2.1 Inhalation Exposure to BEO Can Alleviate Anxiety‐like Behavior in Mice

**Figure 1:**
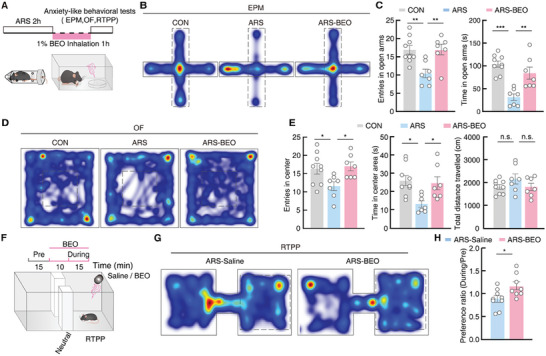
Behavioral effects of 1.0% BEO inhalation at 2 h post‐ARS in mice. A) Experimental paradigm for establishing the acute restraint stress (ARS) mouse model and 1% Bergamot Essential Oil (BEO) inhalation. Stress was induced by enclosing mice for 2 h in a 50 mL plastic syringe with holes drilled for ventilation. Mice were treated with inhalation exposure to 1% BEO (ARS‐1% BEO) or saline control (ARS) for 1 h following restraint. B,C) Heatmaps of locomotion traces from elevated plus maze (EPM) tests B) and summarized data of entries in open arms (C, left) and the time in open arms (C, right) in EPM tests (CON, *n  =*  9 mice; ARS, *n  =*  7 mice; ARS‐1% BEO, *n =* 7 mice; left, F _(2, 20)_ = 16.67, *p* = 0.0026; right, F _(2, 20)_ = 8.319, *p* = 0.0023). D,E) Heatmaps of locomotion traces from open field (OF) tests D) and summarized data of the entries in the center area (E, left), time in center area (E, middle), and total distance traveled (E, right) in OF tests (CON, *n  =*  9 mice; ARS, *n  =*  7 mice; ARS‐1%BEO, *n =* 7 mice; left, F _(2, 20)_ = 4.607, *p* = 0.0226; middle, F _(2, 20)_ = 5.979, *p* = 0.0092; right, F _(2, 20)_ = 1.473, *p* = 0.253). F) Schematic for Real‐time Place Preference (RTPP) tests. (G‐H) Heatmaps of mouse location in RTPP tests and summarized data showing the ratio of time spent in the chamber with BEO or saline before versus during the experimental observation period (ARS‐Saline, *n  =*  9 mice; ARS‐BEO, *n  =*  8 mice; *t*_15_ = 0.0215, *p* = 0.0215). All data are presented as means ± SEM. *p < 0.05, **p < 0.01, ***p < 0.001; n.s., not significant. For detailed statistical information, see also Table S1.

To investigate the effect of BEO on anxiety, we implemented a 2‐h ARS paradigm, during which mice were individually exposed to 0.1% or 1.0% (v/v) BEO or saline in the testing environment, as described previously^[^ ^8^ ^]^ (**Figure** **1**A). Mice treated with no stress (controls), ARS group, or ARS plus 0.1% or 1.0% BEO (ARS‐BEO group) were then examined with the classical paradigm of anxiety‐like behaviors,^[^ ^9^ ^]^ including open‐field (OF) and elevated plus maze (EPM) tests. We found ARS treatment led to decreased exploration time in the center region of the OF and open arms of EPM compared with control mice, suggesting that ARS induced anxiety‐like behaviors in mice (Figure 1B–E), which is consistent with previous studies.^[^ ^10^ ^]^ By contrast, ARS‐1.0% BEO mice spent significantly more time in the center regions and open arms of OF and EPM compared with ARS mice (Figure 1B–E), while ARS‐0.1% BEO showed no difference compared with ARS controls (Figure S1A–E, Supporting Information). These results suggested that inhalation exposure to 1.0% BEO, but not 0.1% BEO, could alleviate ARS‐induced anxiety‐like behaviors in mice, which led us to use the higher concentration in all subsequent experiments. In addition, we found no significant difference in total travel distance among the three groups, indicating that ARS treatment did not affect the mobility of model mice (Figure 1E). Moreover, similar to saline exposure, another odorless placebo control widely used in aromatherapy studies, jojoba oil,^[^ ^11^ ^]^ had no effect on anxiety‐like behaviors in ARS mice (Figure S2A–C, Supporting Information).

In subsequent real‐time place preference (RTPP) tests, ARS mice displayed an obvious preference for the side of the habitat containing the BEO (Figure 1F–H), whereas non‐stress mice showed no preference for the oil side in RTPP tests (Figure S1F–H, Supporting Information). These findings suggested that inhalation exposure to 1% BEO could alleviate ARS‐induced anxiety‐like behaviors in mice.

### 2.2 AON^Glu^ Mediates Anxiolytic‐like Effects Induced by BEO

**Figure 2:**
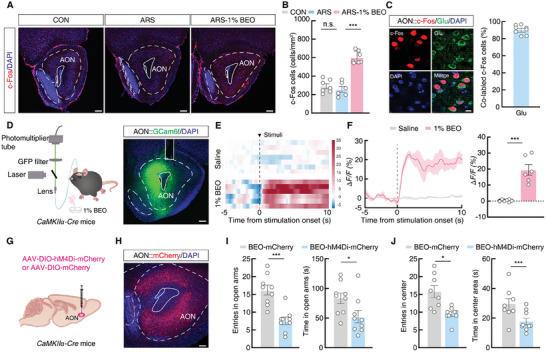
1% BEO mediates anxiolytic effects through AON^CaMKIIα^ neurons in ARS mice. A) Representative images of c‐Fos^+^ neuron distribution in the AON of ARS, ARS‐1% BEO, and no stress (controls) mice. Scale bars, 200 µm. B) Statistical analysis of c‐Fos^+^ neuron distribution in the AON between groups (CON, *n  =*  7 mice; ARS, *n  =*  6 mice; ARS‐1% BEO, *n =* 7 mice; F _(2, 17)_ = 48.95, *p <* 0.0001). C) c‐Fos^+^ neurons co‐localized with glutamatergic neurons in AON. Scale bar, 20 µm. D) Schematic for in vivo fiber photometry recordings (left), and representative image of virus expression and optical fiber implanted above the AON of *CaMKIIα‐Cre* mice. Scale bars, 200 µm. E) Heatmaps across mice aligned to the time from onset of 1% BEO. F) Representative traces (left) and averaged Δ*F/F* (right) of AON^Glu^ signals in Saline and 1% BEO‐treated mice, respectively. The bold line and light shadow indicate mean and SEM, respectively. G,H) Schematic G) and typical image H) for AAV‐DIO‐hM4Di‐mCherry or AAV‐DIO‐mCherry virus injection into the AON of *CaMKIIα‐Cre* mice. Scale bar, 100 µm. I) Statistical summary of entries in open arms (left) and time in open arms (right) for BEO‐mCherry and BEO‐hM4Di‐mCherry mice with 5 mg kg^−1^ CNO injection in EPM tests (BEO‐mCherry, *n  =*  9 mice; BEO‐hM4Di‐mCherry, *n =* 9 mice; left, *t*_16_ = 4.961, *p* = 0.0001; right, *t*_16_ = 2.263, *p* = 0.0379). J) Statistical summary of entries in the center (left) and time in center areas (right), by BEO‐mCherry or BEO‐hM4Di‐mCherry mice with 5 mg kg^−1^ CNO injection in OF tests (BEO‐mCherry, *n  =*  8 mice; BEO‐hM4Di‐mCherry, *n =* 9 mice; left, *t*_15_ = 3.611, *p* = 0.0026; right, *t*_15_ = 2.631, *P =* 0.0189). All data are presented as means ± SEM. *p < 0.05, ***p < 0.001; n.s., not significant. For detailed statistical information, see also Table S1.

The AON is known to play essential roles in odor perception and processing in olfactory‐associated behaviors,^[^ ^6^, ^12^ ^]^ with AON glumatergic neurons (AON^Glu^), in particular, responsible for the output of olfactory information.^[^ ^13^ ^]^ We conducted c‐Fos staining in brain slices of control, ARS, and ARS‐1% BEO mice to first explore the AON neuronal activity after 1% BEO inhalation exposure. Image analysis showed that c‐Fos expression was significantly higher in the AON of ARS‐1% BEO mice than in control or ARS mice (**Figure** **2**A). Further immunofluorescence staining showed that ≈90% of the c‐Fos signal was co‐labeled with a glutamate‐specific antibody (Figure 2B,C).

As the majority of cortical neurons are glutamatergic, and Calcium/calmodulin‐dependent protein kinase II subunit α (CaMKIIα) is widely used as a marker of glutamatergic neurons in the cortex of *CaMKIIα‐Cre* mice^[^ ^14^ ^]^ (although recent studies have shown that CaMKIIα is also expressed in GABAergic neurons^[^ ^15^ ^]^), we next examined AON^CaMKIIα^ neuronal activity in freely moving mice by injecting AAV‐DIO‐GCaMP6f virus and implanting an optical fiber in the AON of *CaMKIIα‐Cre* mice (Figure 2D). Fiber photometry recordings showed that calcium signals rapidly increased in AON^CaMKIIα^ neurons after inhalation of 1.0% BEO, while no significant change was observed following inhalation of the saline vehicle control (Figure 2E,F). Although 0.1% BEO had no significant effect on anxiety‐like behaviors in ARS mice, c‐Fos staining and calcium signal recordings showed that 0.1% BEO exposure could activate AON^CaMKIIα^ neurons, but with a less pronounced effect compared to that of 1% BEO (Figure S3A–F, Supporting Information). These results suggested that BEO inhalation promoted AON^Glu^ neuronal activity in ARS mice. We focused on the AON in further experiments of BEO olfactory processing.

To define the function of AON^Glu^ neurons in the anxiolytic effects associated with BEO inhalation exposure, we injected a Cre‐dependent, inhibitory hM4Di‐mCherry virus (AAV‐DIO‐hM4Di‐mCherry) into the AON of *CaMKIIα‐Cre* mice (which express Cre recombinase in excitatory glutamatergic neurons) (Figure 2G,H; Figure S4A,B, Supporting Information). After 3 weeks, we injected its ligand, clozapine‐N‐oxide (CNO), to inhibit AON^CaMKIIα^ neuronal activity in ARS‐BEO mice. Behavioral tests indicated that ARS‐BEO mice with inhibition of AON^CaMKIIα^ activity spent less time exploring the center region in OF and open arms in EPM compared with ARS‐BEO‐mCherry control mice (Figure 2I,J, Figures S4C and S5A–C, Supporting Information). These results collectively suggested that AON^Glu^ neurons were necessary for processing olfactory information in the development of BEO‐associated anxiolytic‐like effects.

### 2.3 The ACC Receives Direct Inputs from AON^Glu^ Neurons

Olfaction is involved in emotional processing,^[^ ^16^ ^]^ and the AON can actively gate sensory throughput to higher brain centers.^[^ ^17^ ^]^ Therefore, we sought to identify the olfactory‐related neural circuit(s) potentially involved in BEO‐associated modulation of anxiolytic‐like responses. To this end, we searched for brain regions innervated by AON^Glu^ neurons by injecting the AAV‐DIO‐EGFP virus into the AON of *CaMKIIα‐Cre* mice (Figure S6A,B, Supporting Information). In these mice, EGFP terminals originating from the AON could be observed in several brain regions, including the piriform cortex (Pir), medial prefrontal cortex (mPFC), bed nucleus of the stria terminalis (BNST), basal lateral amygdala (BLA), lateral habenula (LHb), and periaqueductal gray (PAG), also in the ACC (Figure S6C, Supporting Information). As the ACC is well‐known to participate in the development of anxiety in humans,^[^ ^18^ ^]^ we therefore focused on the AON→ACC circuit.

**Figure 3:**
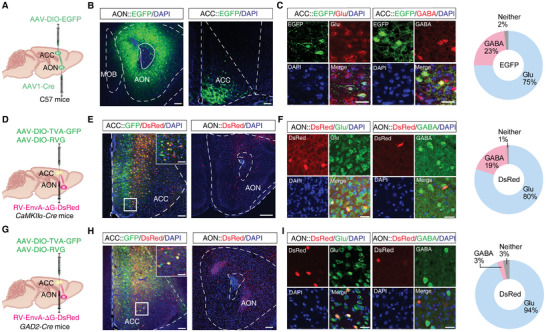
Identification of an AON‐ACC direct projection. A) Schematic for the *Cre*‐dependent anterograde trans‐monosynaptic tracing strategy. B) Representative images of the AAV‐DIO‐EGFP virus injection site in the ACC and AON. Scale bars, 100 µm. C) Representative images (left) and statistical proportions (right) of EGFP^+^ neurons glutamatergic or GABAergic antibody neurons in ACC (*n =* 6 mice per group). D) Schematic for *Cre*‐dependent retrograde trans‐monosynaptic RV tracing strategy in *CaMKIIα‐Cre* mice. E) Representative images of the injection site and virus expression in the ACC of *CaMKIIα‐Cre* mice (left). Starter cells (yellow) co‐express AAV‐DIO‐TVA‐GFP, AAV‐DIO‐RVG (green), and RV‐EnvA‐Δ*G*‐DsRed (red); DsRed‐labeled neurons within the AON (right). Scale bars, 100 µm (ACC), 20 µm (zoom) and 200 µm (AON). F) DsRed‐labeled neurons retrogradely traced from ACC co‐localized with glutamatergic or GABAergic antibody in AON. Scale bars, 20 µm (*n =* 7 mice per group). G) Schematic for *Cre*‐dependent retrograde trans‐monosynaptic RV tracing strategy in *GAD2‐Cre* mice. H) Representative image of the injection site and virus expression within the ACC of *GAD2‐Cre* mice (left). Starter cells (yellow) co‐expressing AAV‐DIO‐TVA‐GFP, AAVDIO‐RVG (green), and RV‐EnvA‐DG‐DsRed (red); DsRed‐labeled neurons within the AON (right). Scale bars, 100 µm (ACC), 20 µm (zoom)and 100 µm (AON). I) DsRed‐labeled neurons traced from ACC that co‐localized with glutamatergic or GABAergic antibody in the AON. Scale bars, 20 µm (*n =* 6 mice per group). All data are presented as mean ± SEM. For detailed statistical information, see also Table S1.

To confirm the anatomical connectivity of this AON→ACC circuit, we conducted anterograde monosynaptic tracing by injecting the AON with AAV‐Cre‐GFP virus while, at the same time, injecting the ipsilateral ACC with AAV‐DIO‐EGFP virus (**Figure** **3**A). After 3 weeks, EGFP^+^ neurons could be observed in the ACC, and immunofluorescence staining showed co‐localization of these EGFP^+^ neurons with signal from glutamatergic antibody (70%), and GABAergic antibody (30%) (Figure 3B,C). Together, these results suggested that both ACC^Glu^ and ACC^GABA^ neurons receive direct projections from the AON.

Next, we used a rabies‐based retrograde monosynaptic tracing strategy by injecting Cre‐dependent helper viruses (AAV‐EF1α‐DIO‐TVA‐GFP and AAV‐EF1α‐DIO‐RVG) into the ACC of *CaMKIIα‐Cre* mice (Figure 3D). After three weeks, an EnvA‐pseudotyped RV‐EnvA‐Δ*G*‐DsRed (RV) was injected at the same site. At one week after RV injection, DsRed^+^ neurons could be observed in several regions, including the AON, that co‐localized with signal for glutamatergic antibody (Figure 3E,F; Figure S7A–D, Supporting Information). We also noted that the DsRed^+^ signal was relatively abundant in the AON, but scarce in the Pir (Figure S8A,B, Supporting Information). In contrast, performing the same tracing strategy in *GAD2‐Cre* mice (which express Cre recombinase in GABAergic neurons) revealed that the DsRed^+^ signal was detectable in the AON and that it colocalized with signal from glutamatergic antibody (Figure 3G,I; Figure S9A,B, Supporting Information). These results confirmed the existence of an AON^Glu^→ACC pathway.

### 2.4 Functional Connections of the AON^Glu^→ACC^GABA→Glu^ Circuit

**Figure 4:**
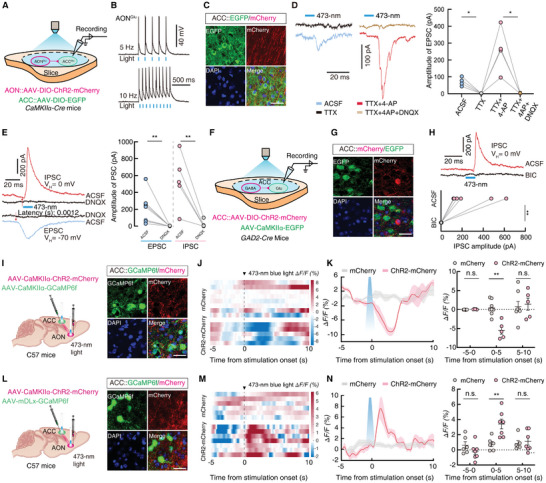
Dissection of the AON^Glu^→ACC^GABA→Glu^ circuit. A) Schematic for optical activation of AON^CaMKIIα^ terminals with simultaneous whole‐cell patch‐clamp recordings of EGFP^+^ ACC^Glu^ neurons. B) Sample traces of action potentials evoked by light (473 nm, 20 ms, blue line) recorded from AON mCherry^+^ neurons in acute brain slices. C) Representative images of the virus expression in the ACC of *CaMKIIα‐Cre* mice. Scale bar, 20 µm. D) Representative traces (left) and summarized data (right) for light‐evoked postsynaptic currents (EPSCs) recorded from ACC^Glu^ neurons (ACSF, *n =* 4 cells, TTX, *n =* 4 cells, *t*_3_ = 5.596, *p* = 0.0113; TTX+4‐AP, *n =* 4 cells, TTX+4‐AP+DNQX, *n =* 4 cells, *t*_3_ = 3.889, *P =* 0.0301). E) Representative traces (left) and summarized data (right) of PSCs recorded from the same ACC^Glu^ neurons after photostimulation of ChR2‐expressing AON^Glu^ terminals in the ACC in brain sections (EPSC, *n =* 8 cells, IPSC, *n =* 5 cells; EPSC, *t*_7_ = 3.059, *p* = 0.0184; IPSC, *t*_4_ = 4.071, *p* = 0.0152). F) Schematic for the optical activation of ACC^GABA^ neurons with simultaneous whole‐cell patch‐clamp recordings of EGFP‐labeled ACC^Glu^ neurons. G) Representative images of the virus expression in the ACC of *GAD2‐Cre* mice. Scale bar, 20 µm. H) Representative traces (top) and summarized data (bottom) of light‐evoked currents (473 nm, 20 ms, blue bar) before and after bicuculline application (IPSC, *n =* 6 cells;t_5_ = 3.221, *p* = 0.0234). I) Schematic for fiber photometry recording of GCaMP6f signal in ACC^CaMKIIα^ neurons upon optogenetic activation of AON^CaMKIIα^ neurons (left); and representative image of GCaMP6f expression in ACC^CaMKIIα^ neurons and ChR2‐expressing axons from AON^CaMKIIα^ neurons (right). Scale bar, 20 µm. J) Heatmaps across trials aligned to the time from photostimulation onset (473 nm, 100 ms, black dashed line). K) Representative traces (left) and averaged Δ*F/F* (right) of ACC^CaMKIIα^ GCaMP6f signals in mCherry and ChR2‐expressing mice. Bold lines and shading indicate mean and SEM, respectively. L) Schematics for fiber photometry recording of GCaMP6f signals in ACC^GABA^ neurons upon optogenetic activation of AON^CaMKIIα^ neurons (left); and representative image of GCaMP6f expression in ACC^GABA^ neurons and axons of ChR2‐expressing AON^Glu^ neurons (right). Scale bar, 20 µm. M) Heatmaps across trials aligned to the time from photostimulation onset (473 nm, 100 ms, black dashed line). N) Representative traces (left) and averaged Δ*F/F* (right) of ACC^GABA^ GCaMP6f signals in mCherry‐ and ChR2‐expressing mice. Bold lines and shading indicate mean and SEM, respectively. All data are presented as means ± SEM. *p < 0.05, **p < 0.01. For detailed statistical information, see also Table S1.

To dissect the functional connections of the AON^Glu^→ACC pathway, we used optogenetic activation of the AON by injecting *Cre*‐dependent ChR2‐mCherry (AAV‐DIO‐ChR2‐mCherry) virus into the AON and AAV‐DIO‐EGFP into the ACC of *CaMKIIα‐Cre* mice (**Figure** **4**A–C). Whole‐cell recordings at ‐70 mV in brain slices of *CaMKIIα‐Cre* mice showed that blue light stimulation of ChR2‐containing AON^CaMKIIα^ terminals in the ACC could reliably elicit excitatory postsynaptic currents (EPSCs). The evoked EPSCs persisted in the presence of tetrodotoxin (TTX) and 4‐aminopyridine (4‐AP) but were blocked by administering the AMPA receptor antagonist, 6,7‐dinitroquinoxaline‐2,3(1H,4H)‐dione (DNQX) (Figure 4D). In addition to the light‐evoked EPSCs, we subsequently observed inhibitory postsynaptic currents (IPSCs) at 0 mV holding potential, which could also be eliminated by exposure to DNQX. We also noted that the latency to the light‐evoked EPSCs (0.0012s) was shorter than that for IPSCs (0.0069s) and that DNQX treatment abolished both EPSCs and IPSCs (Figure 4E). These results suggested a microcircuit organization wherein ACC^Glu^ neurons were innervated by local ACC^GABA^ interneurons, both of which received direct inputs from AON^Glu^ neurons.

To explore the possibility that a microcircuit participates in this olfactory‐associated anxiolytic pathway, we injected AAV‐CaMKIIα‐ChR2‐mCherry virus into the AON and AAV‐DIO‐EGFP virus into the ACC in *GAD2‐Cre* mice (Figure S10A, Supporting Information). Under whole‐cell voltage clamp at ‐70 mV, photostimulation of ChR2‐expressing AON^Glu^ terminals in the ACC reliably evoked EPSCs in ACC^GABA^ neurons, which could be blocked by administering DNQX (Figure S10B,C, Supporting Information). We next injected AAV‐DIO‐ChR2‐mCherry virus and AAV‐CaMKIIα‐EGFP virus into the ACC of *GAD2‐Cre* mice (Figure 4F,G). Blue light stimulation of ACC^GABA^ neurons elicited IPSCs in local glutamatergic neurons, which could be blocked by the administration of the GABA_A_ receptor antagonist, bicuculline (Figure 4H).

To selectively monitor the responses of ACC^Glu^ and ACC^GABA^ neurons after optic activation of AON^Glu^ neurons, we injected AAV‐CaMKIIα‐ChR2‐mCherry virus into the AON, and AAV‐CaMKIIα‐GCaMP6f or AAV‐mDlx‐GCaMP6f virus, respectively, into the ACC of C57 mice (Figure 4I,L). Fiber photometric recordings showed that fluorescence intensity in GCaMP6f‐expressing ACC^CaMKIIα^ neurons was rapidly decreased following photostimulation of ChR2‐expressing AON^CaMKIIα^ neurons. In addition, we observed a rapid and reversible increase in GCaMP6f‐expressing ACC^GABA^ neurons following optical activation of AON^CaMKIIα^ neurons (Figure 4J,K,M,N). These results thus supported the presence of a GABA‐Glu microcircuit in a functional AON^Glu^→ACC^GABA→Glu^ circuit (Figure S10D, Supporting Information).

### 2.5 BEO Inhalation Exposure Activates AON^Glu^→ACC^GABA→Glu^ Circuit

**Figure 5:**
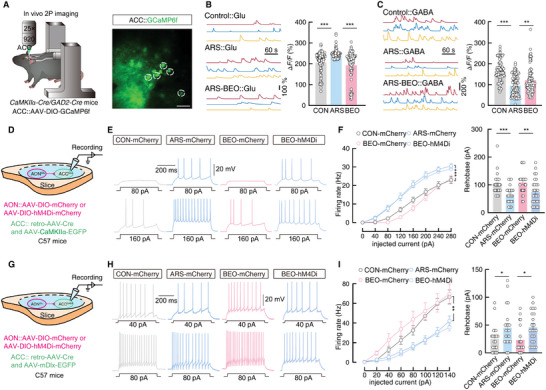
BEO exerts anxiolytic‐like effects by reducing ACC^Glu^ and increasing ACC^GABA^ neuronal activity. A) Schematic of in vivo 2P calcium imaging of ACC neurons in head‐restrained mice (left) and representative image of 2P ACC GCaMP6f imaging field (right). Scale bar, 50 µm. B) Example spontaneous Δ*F/F* time‐series traces from the imaging fields in the panel, average spontaneous calcium responses in GCaMP6f^+^ contralateral ACC^CaMKIIα^ neurons (*n =* 104 neurons per group; F _(2, 309)_ = 21.44, *p <* 0.0001). C) Example spontaneous Δ*F/F* time‐series traces from the imaging fields in panel, average spontaneous calcium responses in GCaMP6f^+^ contralateral ACC^GABA^ neurons (CON, *n =* 100 neurons; ARS, *n =* 100 neurons; ARS‐BEO, *n =* 99 neurons; F _(2, 296)_ = 27.21, *p <* 0.0001). D) Schematic for chemogenetic inhibition of ACC‐projecting AON^CaMKIIα^ neurons and whole‐cell patch‐clamp recordings of EGFP^+^ ACC^Glu^ neurons. (E‐F) Representative traces E) and statistical data F) for action potentials recorded from ACC^CaMKIIα^ neurons of ARS‐BEO mice treated with CNO in the mCherry and hM4Di groups (CON‐mCherry, *n =* 28 neurons; ARS‐mCherry, *n =* 23 neurons; BEO‐mCherry, *n =* 26 neurons; BEO‐hM4Di, *n =* 28 neurons; left, F _(21, 560)_ = 2.429, *p* = 0.0004; right, F _(3, 101)_ = 6.948, *p* = 0.0003). G) Schematic for chemogenetic inhibition of ACC‐projecting AON^CaMKIIα^ neurons and whole‐cell patch‐clamp recordings of EGFP^+^ ACC^GABA^ neurons. H,I) Representative traces H) and statistical data I) for action potentials recorded from ACC^GABA^ neurons of ARS‐BEO mice treated with CNO in the mCherry and hM4Di groups (CON‐mCherry, *n =* 22 neurons; ARS‐mCherry, *n =* 22 neurons; BEO‐mCherry, *n =* 21 neurons; BEO‐hM4Di, *n =* 22 neurons; left, F _(3, 656)_ = 43.12, *p <* 0.0001; right, F _(3, 83)_ = 4.371, *p* = 0.0066). All data are presented as mean ± SEM. *p < 0.05, **p < 0.01, ***p < 0.001. For detailed statistical information, see also Table S1.

We then examined the neuronal activity of this AON^Glu^→ACC^GABA→Glu^ circuit in control, ARS, and ARS‐BEO mice. To this end, we conducted two‐photon calcium imaging in *CaMKIIα‐Cre* mice or *GAD2‐Cre* mice following ACC injection with a *Cre*‐dependent fluorescent Ca^2+^ indicator, GCaMp6f (AAV‐DIO‐GCaMp6f) (**Figure** **5**A; Figure S11A–F, Supporting Information). We found that the fluorescence intensity was significantly increased in ACC^CaMKIIα^ neurons of ARS mice compared with that in control mice, but BEO inhalation exposure could reverse this hyperexcitation of ACC^CaMKIIα^ neurons (Figure 5B; Video S1, Supporting Information). We noted a significant decrease in the fluorescence intensity of ACC^GABA^ interneurons in ARS mice compared with that in control mice but significantly increased in ARS‐BEO mice compared to that in ARS mice (Figure 5C; Video S2, Supporting Information). C‐Fos staining showed that 0.1% BEO had no effect on the number of c‐Fos^+^ cells in the ACC of ARS mice (Figure S12A,B, Supporting Information). In addition, c‐Fos^+^ neurons mainly co‐localized with glutamatergic antibody signal in the ACC of ARS‐0.1% BEO and ARS mice (Figure S12C,D, Supporting Information).

To further verify these trends in AON^Glu^→ACC^GABA→Glu^ circuit activity, we injected AAV‐DIO‐hM4Di‐mCherry or AAV‐DIO‐mCherry into the AON and injected AAV‐CaMKIIα‐EGFP and retro‐AAV‐hSyn‐Cre into the ACC of C57 mice (Figure 5D; Figure S13A,B, Supporting Information). Whole‐cell patch‐clamp recordings in ACC^CaMKIIα^ in brain slices showed that the firing rate decreased and rheobase increased in ARS‐BEO‐mCherry mice compared with that in ARS‐mCherry mice (Figure 5E,F), suggesting that BEO exposure could attenuate the hyperexcitation of this olfactory‐associated stress response pathway. Alternatively, ACC^CaMKIIα^ neurons showed an increase in firing rate and a decrease in rheobase in ARS‐BEO‐hM4Di‐mCherry mice compared with that in ARS‐BEO‐mCherry controls (Figure 5E,F), which suggested that inhibiting this pathway could block the effects of BEO on this pathway. We also performed whole‐cell patch‐clamp recordings of ACC^GABA^ neurons in brain slices of C57 mice injected with AAV‐DIO‐hM4Di‐mCherry or AAV‐DIO‐mCherry in the AON and AAV‐mDlx‐EGFP and retro‐AAV‐hSyn‐Cre in the ACC (Figure 5G; Figure S13C,D, Supporting Information). We observed that the ACC^GABA^ interneurons showed an increase in firing rate and a decrease in the rheobase in ARS‐BEO‐mCherry mice compared with that in ARS‐mCherry mice (Figure 5H,I), indicating that BEO exposure is associated with increased inhibitory activity in this circuit. Conversely, ACC^GABA^ firing rate decreased and rheobase increased in ARS‐BEO‐hM4Di‐mCherry mice compared with the corresponding ARS‐BEO‐mCherry controls (Figure 5H,I). Given the increased ACC^GABA^ activity in response to BEO in ARS mice, we selectively inhibited ACC^GABA^ interneurons through injection of AAV‐DIO‐hM4Di‐mCherry in mice, which resulted in blocking the BEO‐induced alleviation of anxiety‐like behaviors (Figure S14A–D, Supporting Information). Taken together, these results demonstrated that BEO activates the AON^Glu^→ACC^GABA→Glu^ circuit, which can inhibit ACC^Glu^ excitation through a local GABA→Glu microcircuit.

### 2.6 The AON^Glu^→ACC^GABA→Glu^ Circuit Mediates Anxiolytic‐like Effect Induced by BEO

**Figure 6:**
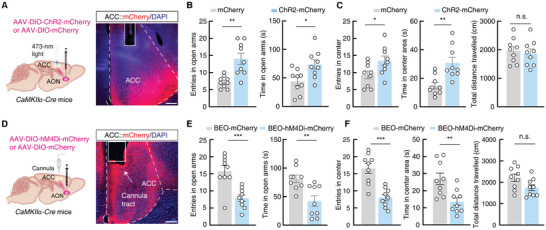
The AON^Glu^→ACC circuit controls anxiolytic‐like effects induced by BEO. A) Schematic for AAV‐DIO‐ChR2‐mCherry or AAV‐DIO‐mCherry virus injection into the AON of *CaMKIIα‐Cre* mice and representative image of virus expression and optical fiber implanted above the ACC of *CaMKIIα‐Cre* mice. Scale bar, 100 µm. B) Summarized data of entries in open arms (left) and time in open arms (right) by mCherry control or ChR2‐mCherry mice in EPM tests (mCherry, *n  =*  9 mice; ChR2‐mCherry, *n =* 9 mice; left, *t*_16_ = 3.954, *p* = 0.0011; right, *t*_16_ = 2.753, *p* = 0.0141). C) Summarized data of entries in center (left), time in center areas (middle), and total distance traveled (right) by mCherry control or ChR2‐mCherry mice in OF tests (mCherry, *n  =*  9 mice; ChR2‐mCherry, *n =* 9 mice; left, *t*_16_ = 2.299, *p* = 0.0353; middle, *t*_16_ = 3.590, *p* = 0.0025; right, *t*_16_ = 1.102, *p* = 0.2869). D) Schematic for AAV‐DIO‐hM4Di‐mCherry or AAV‐DIO‐mCherry virus injection into the AON of *CaMKIIα‐Cre* mice. Representative image of virus expression and cannula tract in the ACC of *CaMKIIα‐Cre* mice. Scale bar, 100 µm. E) Summarized data of entries in open arms (left) and time in open arms (right) by BEO‐mCherry or BEO‐hM4Di‐mCherry mice in EPM tests (BEO‐mCherry, *n  =*  9 mice; BEO‐hM4Di‐mCherry, *n =* 9 mice; left, *t*_16_ = 4.180, *p* = 0.0007; right, *t*_16_ = 3.504, *p* = 0.0029). F) Summarized data of entries in center (left), time in center areas (middle), and total distance traveled (right) by BEO‐mCherry or BEO‐hM4Di‐mCherry mice in OF tests (BEO‐mCherry, *n  =*  9 mice; BEO‐hM4Di‐mCherry, *n =* 9 mice; left, *t*_16_ = 4.960, *p* = 0.0001; middle, *t*_16_ = 3.275, *p* = 0.0048; right, *t*_16_ = 1.750, *p* = 0.0992). All data are presented as means ± SEM. *p < 0.05, **p < 0.01, ***p < 0.001; n.s., not significant. For detailed statistical information, see also Table S1.

We then explored whether activation of this AON^Glu^→ACC^GABA→Glu^ circuit could alleviate the anxiety‐like effects of ARS, thus recapitulating the response to BEO observed in ARS mice. To test this possibility, we first injected AAV‐DIO‐ChR2‐mCherry virus into the AON of *CaMKIIα‐Cre* mice and ipsilaterally implanted optic fibers above the ACC region (**Figure** **6**A). We found that optical activation of ChR2‐expressing AON^CaMKIIα^ terminals in the ACC led to reduced anxiety‐like behavior in ARS mice, indicated by more time in the center and open arms of the OF and EPM compared with that of ARS‐mCherry control mice (Figure 6B,C). We subsequently injected AAV‐DIO‐ChR2‐mCherry virus and implanted optical fibers into the ACC of *GAD2‐Cre* mice, which led to a similar reduction in anxiety‐like behaviors upon photostimulation of ChR2‐expressing ACC^GABA^ terminals in the ACC (Figure S15A–D, Supporting Information). These results collectively suggested that activation of the AON^Glu^→ACC^GABA→Glu^ circuit indeed reduces anxiety‐like behaviors associated with ARS.

We then sought to verify whether inhibiting this pathway could block the anxiolytic‐like effects of BEO by injecting the bilateral AON with AAV‐DIO‐hM4Di‐mCherry and implanting cannulas in the ACC of *CaMKIIα‐Cre* mice (Figure 6D). Intracranial microinjection of CNO in the ACC was applied to inhibit ACC neurons innervated by the AON. We found that ARS‐BEO‐hM4Di‐mCherry mice, with inhibition of AON^CaMKIIα^ terminals in the ACC, exhibited shorter time in the center of the OF and open arms of EPM compared with ARS‐BEO‐mCherry control mice (Figure 6E,F), indicating that inhibition of these BEO‐responsive AON^Glu^ projections could block the anxiolytic effects of BEO. And cumulatively demonstrating that the AON^Glu^→ACC^GABA→Glu^ circuit is sufficient and necessary for anxiolytic‐like effect induced by BEO (Figure S16, Supporting Information).

## 3 Discussion

It is well‐established that olfactory perception can profoundly impact an individual's daily emotional state, suggesting that aromatherapy can alleviate negative emotions, in addition to its known effects in relieving pain and promoting sleep.^[^ ^17^, ^19^ ^]^ The present study defines a previously undocumented AON^Glu^→ACC^GABA→Glu^ neural circuit that mediates anxiolytic effects in response to inhalation of BEO in mice. We found that activation of ACC^Glu^ neurons can increase anxiety‐like behaviors, whereas activation of ACC^GABA^ interneurons suppresses anxiety‐like behaviors. This pathway from AON to ACC is activated following BEO exposure, and ACC^GABA^ interneurons could inhibit ACC^Glu^ neurons through local microcircuit connections.

RTPP tests are commonly employed to assess positive or negative responses to odors of mice.^[^ ^20^ ^]^ Our results demonstrated that non‐stress control mice showed no preference for the BEO side, indicating that mice had no preference or aversion to BEO odor prior to inducing stress. However, following ARS induction, mice exhibited a significant preference for the BEO side. In our study, ARS mice exhibited anxiety‐like behaviors that could be alleviated by BEO inhalation exposure. In the absence of evidence demonstrating that ARS could alter olfactory preference in mice, and in light of our experimental results, we hypothesized that the observed preference for BEO odor observed in ARS mice may be due to its effects in alleviating anxiety‐like behavior.

Notably, we observed comparable fluorescence intensity in the Pir and ACC after injecting the AAV‐DIO‐EGFP virus into the AON of *CaMKIIα‐Cre* mice. The Pir is the classic downstream nucleus of the AON.^[^ ^6^ ^]^ Olfactory signals are initially received by the OB, and then subsequently transmitted to the AON, and thus the AON serves as a conduit for transmitting olfactory information to the Pir and other brain regions involved in olfactory processing. This study aimed to reveal the neural circuit between olfactory perception and emotional processing underlying BEO‐induced anxiolytic effects. As the Pir is an olfactory‐related brain region,^[^ ^12^ ^]^ while the ACC is closely linked to emotional responses,^[^ ^21^ ^]^ together with previous EEG data demonstrating that essential oil inhalation can facilitate cortical activity in humans, including the ACC,^[^ ^7^, ^22^ ^]^ we selected the ACC for further investigation of its role in linking olfaction with emotional response. However, based on our results, we cannot exclude the possibility that Pir may exert its effects on downstream brain regions associated with emotional processes through direct or indirect circuit pathways.

The ACC has been well‐established as a key region involved in emotional responses and sensory perception,^[^ ^23^ ^]^ and is considered a primary neural target of stress. In addition, the ACC mediates various aspects of stress‐related physiology and pathology,^[^ ^24^ ^]^ such as stress‐induced hyperalgesia, fear, and anxiety.^[^ ^21^, ^25^ ^]^ Hyperactivity of the ACC has been observed in both rodent models and human patients with anxiety, whereas inactivation of the ACC can relieve anxiety.^[^ ^26^ ^]^ Interestingly, we found that different types of neurons in the ACC exert opposite effects on stress‐induced anxiety. Specifically, ACC^Glu^ neurons exhibited increased excitation in our ARS model mice, whereas activity decreased in ACC^GABA^ neurons. In addition, we found that inhalation of 1.0% BEO could elicit significant anxiolytic effects through activation of an inhibitory ACC^GABA→Glu^ microcircuit. Although previous studies and our viral tracing results have shown that several brain regions are innervated by the olfactory system,^[^ ^27^ ^]^ our current study implies that the ACC could be a critical brain region for the olfactory system to regulate the emotional system.

Previous studies have suggested a close relationship between the olfactory system and emotional information processing, and some odors have been shown to modulate mood, including depression and anxiety.^[^ ^16^ ^]^ The olfactory system is linked to the limbic system, thus reinforcing the interplay between emotion and cognition.^[^ ^28^ ^]^ In addition to the olfactory system, the visual and auditory systems can also play significant roles in modulating emotional response.^[^ ^25^, ^29^ ^]^ Crosstalk between these different systems may serve as the basis for multi‐system regulation of emotional disorders. Aromatic essential oils thus show strong potential as widely accessible, inexpensive, and side‐effect‐free complementary physical therapies.

Previous preclinical studies have explored the neuronal mechanisms underlying the therapeutic efficacy of essential oils in alleviating anxiety. For example, essential oil treatment can increase blood 5‐HT concentrations in elderly people.^[^ ^30^ ^]^ Similarly, salivary cortisol levels were observed to decline following BEO vapor inhalation in female volunteers relative to those inhaling water vapor.^[^ ^31^ ^]^ In addition, EEG recordings have demonstrated that inhalation exposure to some essential oils can stimulate cortical activity in humans, including the ACC.^[^ ^7^, ^22^ ^]^ These findings suggest that inhaling volatile compounds in essential oils could induce anxiolytic effects through the central nervous system (CNS). However, the neural circuit mechanisms through which essential oil inhalation could induce anxiolytic effects have remained elusive. Our current study uncovered a cell‐type‐specific neural circuit mediating the anxiolytic effects induced by inhalation exposure to BEO.

Inhalation and oral administration are frequently employed methods for administering essential oils in preclinical and clinical trials,^[^ ^32^ ^]^ with skin application more commonly used in humans, and intraperitoneal injection generally used in animal models.^[^ ^32^ ^]^ In addition to the olfactory signal pathway, essential oils can be absorbed into blood through the lungs and nasal mucosa.^[^ ^32^ ^]^ Previous studies have demonstrated that components of essential oils can be detected in both blood and brain tissues following inhalation.^[^ ^32^, ^33^ ^]^ Oral or intraperitoneal administration of essential oils can modulate neuronal activity in the hippocampus,^[^ ^34^ ^]^ also it is noteworthy that anosmia was not found to interfere with the anxiolytic effect of lavender essential oil^[^ ^35^ ^]^ or Valerena‐4,7(11)‐diene.^[^ ^36^ ^]^ These studies collectively indicate that bioactive compounds in essential oils may reach systemic circulation through different administration methods, and modulate emotional response pathways in the CNS.

Previous studies have shown that different stress models (e.g., immobilization stress, pharyngeal inflammation, chronic social defeat stress) induce anxiety‐like or depression‐like behaviors via different neural circuits,^[^ ^37^ ^]^ and each respective circuit mediating a distinct depression‐like behavioral phenotype (e.g., social withdrawal or despair behaviors).^[^ ^38^ ^]^ This broad involvement of multiple independent circuits in mediating anxiety or depression behaviors in response to different etiologies thus presents a challenge for identifying clinical treatment strategies that can effectively target specific stress response pathways or behavioral disorders. In the present study, inhalation of BEO was employed as a therapeutic modality for alleviating anxiety. It is possible that essential oils exert anxiolytic or antidepressant effects by widely influencing multiple brain regions through the olfactory or circulatory system. Identifying the various neural circuit mechanisms through which essential oils alleviate anxiety or depression expands our understanding of the neural mechanisms underlying emotional disorders.

In summary, this study expands our understanding of the conventional anxiety processing pathway by characterizing the influence of BEO on anxiety. Findings in this work can thus accelerate research into the role of odors in regulating anxiety and other emotions and suggest potential targets for therapeutic development to treat anxiety‐related disorders. Our study also provides a rational basis for the further exploration of BEO aromatherapy as a non‐pharmacological, complementary medicine for treating anxiety and potentially other mood disorders.

## 4 Experimental Section

### Animals

Male mice aged 8–10 weeks were used for all experiments. All mice were purchased from Charles River or Jackson Laboratories including C57BL/6J, *CaMKIIα‐Cre*, and *GAD2‐Cre* mice. Five mice were housed in one cage with ad libitum access to get enough water and food. They were housed at a stable temperature (23–25 °C) with a 12 h light‐dark cycle (lights on from 7:00 a.m. to 7:00 p.m.). All animal experiments were approved by the Animal Care and Use Committee of the University of Science and Technology of China (USTCACUC26080123082).

### Acute Restraint Stress (ARS) Model

Mice were exposed to acute restraint stress by being confined in a 50‐mL plastic syringe with some holes drilled to allow breath for 2 hours. The control mice were deprived of food and water during the same period but were allowed to move freely in their cages. The behavioral tests were conducted after resting for 1 h.

### Inhalation of Bergamot Essential Oil (BEO)

Chromatographic results of BEO (Oshadhi, Germany) on the certificate of analysis provide the following composition of the batch: d‐limonene, 39.60%; linalyl acetate, 31.09%; and linalool, 9.55%. 1% or 0.1% BEO was diluted with saline and was freshly prepared on the day of the experiment.

To effectively evaporate BEO or saline and jojoba oil, cotton soaked with saline or BEO and jojoba oil was placed into a culture dish (diameter of 60 mm) with some pores. The culture dishes were placed in transparent cages (484 cm^2^ × 13.5 cm length) for 1 h, which were covered with filter paper, allowing air to pass through. After inhaling BEO, no mice exhibited abnormal behavior, including increased or decreased movement, and loss of body posture.

### Behavioral Tests

Mice were placed in the behavioral test room 3 days in advance to acclimatize for at least 2 hours each time, and the tester stroked the mice for 1–2 minutes each day to relieve their anxiety. During behavioral tests, mice were placed under dim light (≈20 lux) and their behavior was recorded using behavioral video. At the end of each experiment, the behavioral apparatus was thoroughly cleaned with 75% ethanol and rinsed with pure water to eliminate scents that might interfere with experimental results. Video analysis was performed using EthoVision XT 14 software (Noldus, the Netherlands).

### Real‐time Place Preference (RTPP) Test

An apparatus consisting of two chambers (40 × 20 cm) connected by a “neck” structure was used for RTPP tests. Before the experiment, mice moved around two chambers for 10 minutes on day 1 to determine the preferred or non‐preferred compartments. If a mouse spent more than 60% of the 10 minutes in either compartment, it was excluded from further experiments.

For a real‐time olfactory preference experiment on BEO, the experiment was divided into two parts. In the first part, 1 mL of saline was placed in two chambers respectively and mice were allowed to freely move in both chambers. A 15 min pre‐treatment experiment first (Pre) was conducted, followed by 10 minutes of odor dissipation, and finally, a 15 min formal experiment (During) was conducted. In the second part, choose one chamber as the stimulation chamber to place 1 mL 1% BEO diluted with saline, and the other chamber to place 1 mL saline, allowing mice to freely move in two chambers. The preference ratio was calculated by dividing the time spent in the During period by that in the Pre period.

### Open Field (OF) Test

The instrument was mainly made of a white single‐sided frosted 50 × 50 × 60 cm^3^ acrylic chamber with a central area of 25 × 25 cm^2^. Mice were carefully placed in the central area and their movements were recorded during the first 6 minutes, EthoVision XT 14 software was used to analyze the last five minutes of movement.

### Elevated Plus Maze (EPM) Test

The EPM consists of two open arms and two closed arms, with a size of 30 × 6 × 20 cm^3^, they intersect vertically with each other, forming a size 6 × 6 cm^2^ central area platform. The maze was 100 cm above the ground. During the experiment, the mice were gently placed in the central area with their open arms. Behavioral recording software was used to record the first 6 minutes after the start of the assay, and EthoVision XT 14 software was used to analyze the last five minutes of movement.

### Immunofluorescence, Imaging, and Image Analysis

The mice were first quickly anesthetized with isoflurane followed by an intraperitoneal injection of pentobarbital sodium (20 mg kg^−1^). After the mice were completely anesthetized, they were sequentially perfused with saline and 4% (w/v) paraformaldehyde (PFA). The brain of the mouse was taken out and placed in a 10 mL tube containing 4%PFA for 10–12 hours. Then, the brain was sequentially placed in a 20% and 30% sucrose solution overnight until it sank to the bottom. For immunofluorescence, 40 µm coronal sections were cut using a frozen sectioning machine (RWD, China). First, brain slices were washed with PBS three times. Then, PBS containing 0.3% (w/v) Triton X‐100 and 3% donkey serum was used for blocking. After blocking for 1 h, the brain slices were stained with a primary antibody. The antibodies include anti‐GABA (1:500, rabbit, Cat# A2052, Sigma, USA), anti‐glutamate (1:500, rabbit, Cat# G6642, Sigma, USA), anti‐c‐Fos (1:500, rabbit, Cat# 226008, Synaptic Systems, USA). The diluent of the primary antibody included corresponding antibodies, 0.3% BSA, 0.3% Triton X, and 3% donkey serum, and brain slices were incubated at 4 °C for 24 hours. After the incubation of the first antibody, the brain slices were incubated with fluorophore‐conjugated Alexa Fluor 488 and Alexa Fluor 594 for one and a half hours. Then, the brain slices were washed three times with PBS and stained with DAPI.

Fluorescence signals were detected using FV3000 (Olympus, Japan), Zeiss LSM880 and LSM980 microscopes. ImageJ software (NIH) was used to calculate c‐Fos, the colocalization of Glu and GABA with c‐Fos. The fluorescent intensity of presynaptic terminals originating from AON^Glu^ neurons was quantified using ImageJ software. Specifically, each brain section was transformed into an 8‐bit image, and the brain regions of interest were delineated manually according to the brain atlas. Subsequently, the fluorescence density was calculated by counting the sum of the grey values of all pixels within the selection and dividing by the number of pixels. The axon density in each brain structure was normalized to the average fluorescence density in the Pir from AON^Glu^ neurons.

### Stereotaxic Surgery and Virus Injection

The mice were anesthetized through intraperitoneal injection of pentobarbital (20 mg kg^−1^) prior to surgery. They were then placed on a stereotaxic instrument (RWD, China) and immobilized while their central body temperature was kept at 36 °C using a heating pad. After sterilization and a midline scalp incision, the skull surface was exposed and leveled. The coordinates (in mm) were defined for the anterior‐posterior (AP), longitudinal (ML), and dorsoventral (DV) aspects of the anterior fontanelle and the brain surface. A small craniotomy (0.5 mm) was made using a syringe tip and adjustable speed dental drill (B67275, Meisinger, Germany). The virus was then injected into the brain region specifically at a speed of 30 nL min^−1^ using a 10 mL microsyringe with a calibrated glass microelectrode and syringe pump (1B 100–3, WPI, USA). To avoid virus leakage, the microsyringe was left at the injection site for 5–10 min after the virus injection was completed. Finally, the incision was sutured and the surgical wound was sterilized.

### Optogenetic Manipulation

For optogenetic manipulation, the *Cre*‐dependent virus AAV‐DIO‐ChR2‐mCherry (rAAV‐Ef1α‐DIO‐hChR2 (H134R)‐mCherry‐WPRE‐pA, AAV2/5, 5.2 × 10^12^ vgmL^−1^, 200 nL) was delivered into the AON (AP: +2.05 mm; ML: −1.0 mm; DV: −2.98 mm) of *CaMKIIα‐Cre* mice and ACC (AP, +0.38 mm; ML, −0.25 mm; DV, −1.12 mm) of *GAD2‐Cre* mice. Optical fibers (200 µm OD, 0.37 NA, Inper) were implanted in the ACC.

After three weeks of virus expression, the optical fibers were connected to the photogenetic equipment through jumpers, fiber rotators, fibers, and ceramic sleeves (RWD, China). Then, the mice were placed back in the breeding cage for at least 30 minutes. Next, blue light (473 nm, 5–8 mW, 15‐ms pulses, 20 Hz), controlled by the Master‐8 pulse stimulator (a.M.P.I., Israel), was delivered to selectively activate the AON^Glu^ terminals or ACC^GABA^ terminals in the ACC.

### Chemogenetic Manipulation

For chemogenetic manipulation, the AAV‐DIO‐hM4Di‐mCherry (rAAV‐Ef1α‐DIO‐hM4D(Gi)‐mCherry‐WPRE‐pA, AAV2/9, 2.25 × 10^12^ vgmL^−1^, 200 nL) virus was injected in the AON of *CaMKIIα‐Cre* mice. The AAV‐DIO‐mCherry (rAAV‐Ef1α‐DIO‐mCherry‐WPRE‐pA, AAV2/9, 5.31 × 10^12^ vgmL^−1^, 200 nL) virus was used as the control. For chemogenetic inhibition of AON^Glu^, the chemical ligand CNO (1 mg kg^−1^ or 5 mg kg^−1^, HY‐17366, Sigma, USA) was intraperitoneally injected under isoflurane anesthesia. Behavioral tests were conducted at least 30 minutes later.

For chemogenetic inhibition of AON^Glu^ terminals in ACC, the drug administration cannula (0.25 mm inner diameter, RWD, China) was implanted into the ACC. CNO (3 µm, 100 nL) was intracranial microinjection by syringe pump. Behavioral tests were conducted at least 30 minutes later. The control group received the same regimen. After completing all behavioral tests, the mice were perfused to verify the virus injection site and cannula site.

### Fiber Photometry

Calcium signals were recorded using fiber photometry, the AAV‐DIO‐GCaMP6f (AAV‐DIO‐GCaMP6f, AAV2/9, 2.53 × 10^12^ vgmL^−1^, 200 nL) was injected and an optic fiber was implanted into the AON of *CaMKIIα‐Cre* mice. The GCaMP6f fluorescence intensity of AON^Glu^ was recorded when the cotton containing BEO or saline was placed in the mouse cages. Light from a 470‐nm LED (3 ms, 40 Hz) was bandpass filtered (470/10 nm), collimated, reflected by dichroic mirrors (MD498, Thorlabs), and coupled to an optic commutator (Doris Lenses) after focusing with an objective lens (0.4 NA, Olympus). Light stimulation was then delivered at a power of 25–40 mW at the tip of the optic fiber to excite GCaMP6f fluorescence. The emitted fluorescence from GCaMP6f was then bandpass filtered (525/40 nm, Thorlabs) and focused on the sensor of a CMOS camera. The end of the fiber was imaged at a frame rate of 40 fps with InperSignal, and the mean value of the ROI at the end‐face of the fiber was calculated using InperPlot software. The values of fluorescence change (Δ*F/F)* were derived by calculating Δ*F/F* (%)  =  (F(duration) ‐ F(baseline)/F(baseline)) × 100%, and the signals at 5 s before stimulus presentation were defined as the baseline.

To record the calcium signals of ACC^Glu^ and ACC^GABA^ neurons after optic activation of AON^Glu^ neurons, the AAV‐CaMKIIα‐ChR2‐mCherry (rAAV‐CaMKIIα‐ChR2‐mCherry‐WPRE‐hGH pA, AAV2/9, 5.14 × 10^12^ vgmL^−1^, 200 nL) virus was injected into the AON, and the AAV‐CaMKIIα‐GCaMP6f or AAV‐mDlx‐GCaMP6f (rAAV‐mDlx‐GCaMP6f‐WPRE‐pA, AAV2/9, 2 × 10^12^ vgmL^−1^, 200 nL) virus was injected into the ACC, and an optic fiber was implanted above the ACC of C57 mice. Calcium signals of ACC neurons were recorded before and after 473 nm blue light stimulation (20 ms). The time of stimulation was defined as 0 s. The mean values of Δ*F/F* during the intervals of −5‐0 s, 0–5 s, and 5–10 s were calculated to reflect ACC neural activity before stimulation, in response to light, and after light response, respectively.

### In vitro Electrophysiological Recordings—Brain Slice Preparation

Mice were deeply anesthetized with pentobarbital sodium (2%, w/v, i.p.) and after intracardial perfusion with 20 mL oxygenated NMDG ACSF, which included (in mM): 93 NMDG, 2.5 KCl, 1.2 NaH_2_PO_4_, 25 glucose, 30 NaHCO_3_, 5 Na‐ascorbate, 20 HEPES, 2 thiourea, 10 MgSO_4_, 2.5 KCl, 3 glutathione (GSH) and 2 CaCl_2_·2H_2_O (pH:7.3‐7.4, osmolarity: 300–305 mOsm kg^−1^). Coronal slices (270‐300 µm) containing ACC were sectioned with a vibratome (Leica VT1200s, Germany), After that, the brain slices were initially incubated in oxygenated NMDG ACSF for 10 min at 32 °C. Then slices were moved to oxygenated HEPES ACSF, which included (in mM): 20 HEPES, 2.5 KCl,1.2 NaH_2_PO_4_, 25 glucose, 92 NaCl, 30 NaHCO_3_, 2 thiourea, 3 Na‐pyruvate, 3 glutathione(GSH), 2CaCl_2_·2H_2_O, 2 MgSO_4_ and 5 Na‐ascorbate (28 °C, pH:7.3‐7.4, osmolarity: 300 −305 mOsm kg^−1^) for at least 1 h. The brain slices were then transferred to a slice chamber (Warner Instruments, USA) for whole‐cell recording and slowly perfused (3 mL min^–1^) with oxygenated standard ACSF solution (32 °C, pH: 7.3‐7.4, osmolarity: 300–310 mOsm kg^−1^), which included (in mM) 3 HEPES, 10 glucose, 129 NaCl, 3 KCl, 1.2 KH2PO_4_, 1.3 MgSO_4_, 2CaCl_2_·2H_2_Oand 20 NaHCO_3_.

### In vitro Electrophysiological Recordings—Whole‐Cell Patch Clamp Recordings

Whole‐cell patch‐clamp recordings were performed using an infrared differential interference contrast microscope (BX51WI, Olympus, Japan) equipped with interference contrast (IR/DIC) and an infrared camera connected to the video monitor. Recording pipettes (3‐5 MΩ) were pulled from borosilicate glass capillaries (VitalSense Scientific, China) with an outer diameter of 1.5 mm on a four‐stage horizontal puller (P‐1000, Sutter, USA). A MultiClamp 700B amplifier and pCLAMP10.7 software were applied to collect electrophysiological signals. After a stable Gigaseal was formed, the capacitance and series resistance were automatically compensated. Current‐evoked firings in GABA and Glu of ACC neurons were recorded separately under current‐clamp mode (I = 0 pA) and under voltage‐clamp mode (V_H_ = −70 mV) by using pipettes (5–8 MΩ) filled with potassium‐gluconate‐based internal resistance solution, which include 130 potassium gluconate, 2 MgCl_2_, 5 KCl, 0.6 EGTA, 10 HEPES, 2 Mg‐ATP and 0.3 Na‐GTP (pH: 7.2, osmolality: 285–290m Osm kg^−1^).

### In vitro Electrophysiological Recordings—Light‐evoked Response

To verify the functional properties of the AAV‐DIO‐ChR2‐mCherry virus, AAV‐DIO‐ChR2‐mCherry was injected into the AON, and AAV‐CaMKIIα‐EGFP was injected into the ACC. mCherry‐labeled neurons expressing ChR2 in the ACC were visualized and subjected to 5 Hz and 10 Hz blue laser stimulation (473 nm, 5–10 mV) with a pulse width of 15 ms. EPSCs were recorded at −70 mV after photostimulation of ChR2‐expressing AON^Glu^ fibers in ACC slices (473 nm, 10 V, 20 ms). IPSCs were recorded at 0 mV after photostimulation of ChR2‐expressing AON^Glu^ fibers or ACC^GABA^ fibers in ACC slices (473 nm, 10 V, 20 ms). The EPSCs and IPSCs were blocked by 10 µm DNQX (D0540, Sigma, USA) injected with ACSF. To test whether the postsynaptic currents recorded in ACC^Glu^ neurons were elicited by direct synaptic connections, 1 µm tetro‐dotoxin (TTX, Hebei Aquatic Science and Technology Development Company, China) and 1 µm 4‐aminopyridine (4‐AP) were added to the ACSF.

To explore the microcircuit, AAV‐DIO‐ChR2‐mCherry virus, and AAV‐CaMKIIα‐EGFP virus were injected into the ACC of *GAD2‐Cre* mice, IPSCs were recorded at 0 mV after photostimulation of ChR2‐expressing ACC^GABA^ fibers in ACC slices (473 nm, 10 V, 20 ms). The IPSCs were blocked by 100 µm BIC (BIC, O7639, Sigma) injected with ACSF.

To record AON^Glu^‐innervated ACC neurons, AAV‐DIO‐hM4Di‐mCherry or AAV‐DIO‐mCherry was injected into the AON, and AAV‐CaMKIIα‐EGFP or AAV‐mDlx‐EGFP and retro‐AAV‐hSyn‐Cre was injected into the ACC of C57 mice, neuronal excitability of the ACC^Glu^ and ACC^GABA^ neurons were recorded.

### In vivo 2P Calcium Imaging—Cranial Window Surgery

Mice were anesthetized through intraperitoneal injection of pentobarbital (20 mg kg^−1^) prior to surgery. Mice were immobilized on the stereotaxic instrument. Next, a solution of 2% iodophor and 75% alcohol was used as a disinfectant in the targeted brain region of the *CaMKIIα‐Cre* or *GAD2‐Cre* mice. The scalp and periosteum covering the dorsal skull were removed. A 3 × 3 mm piece of skull was removed with a dental drill and positioned on the ACC according to the stereotactic coordinates. Following craniectomy, the AAV‐DIO‐GCaMP6f virus was in the ACC. After that, a smaller circular coverslip (3 mm, Bellco Glass Inc.) was introduced, and the region was covered with 1% agarose. Finally, dental adhesive and 3M tissue adhesive were used to attach a specially‐made aluminum head plate to the skull. Dexamethasone (5 mg kg^−1^, HY‐14686, MedChemExpress, USA) and Enrofloxacin (1 mg kg^−1^, HY‐B0502, MedChemExpress, USA) were administrated once before surgery for a week.

### In vivo 2P Calcium Imaging—Calcium Imaging

The mice were allowed to recover from cranial window surgery for 2–4 weeks and needed to be adapted for 3 days, 20 min a day before imaging. In vivo 2P calcium imaging was performed on an upright 2P microscope (FVMPE‐RS, Olympus, Japan) coupled with a Mai Tai Deep See laser (Spectra‐Physics) and scanning galvanometer. The laser operating wavelength was 920 nm (10% laser transmissivity and ≈380 V of PMT voltage) for GCaMP6f, with 30 mW average power on the sample.

### In vivo 2P Calcium Imaging—Data Processing and Analysis

Time‐series data were exported to ImageJ for further analysis.

Imaging data were corrected for mechanical drift with the TurboReg plugin, which can be found at http://bigwww.epfl.ch/thevenaz/turboreg/. Time‐lapse videos were obtained by using sequential images. These corrected data were analyzed using an open‐source routine, which was available via GitHub (https://github.com/flatironinstitute/CaImAn‐MATLAB). The calcium images were segmented into individual cells using MATLAB scripts, with component extraction based on pre‐specified descriptions of the spatial footprint (location and shape) and the activity trace of the source. Traces of Δ*F/F* versus time were automatically generated for each GCaMP6f‐expressing neuron.

### Statistical Analysis

Animals of consistent age were randomized to exclude experimental errors caused by objective factors. All data were presented as mean ± SEM. Student's *t*‐test was used for simple statistical comparisons with significance levels of *p < 0.05, **p < 0.01, and ***p < 0.001. One‐way or two‐way analysis of variance (ANOVA) and Bonferroni *post hoc* analyses were used in analyses with multiple experimental groups. GraphPad Prism 9 (GraphPad Software, USA) was used for analysis and graphing. Clampfit software version 10.6 (Axon Instruments, USA) was used to analyze data from electrophysiological experiments offline.

All statistical data, significance analysis, number of individual experiments (*n*), and other relevant information used for data comparison were described in Table S1, Supporting Information.

## Conflict of Interest

The authors declare no conflict of interest.

## Supporting information



Supporting Information

Supplemental Video 1

Supplemental Video 2

## Data Availability

The data that support the findings of this study are available from the corresponding author upon reasonable request.
