# Supplementary material for: A Neural Circuit For Bergamot Essential Oil‐Induced Anxiolytic Effects
Source: Adv Sci (Weinh). 2024 Nov 2;12(1):2406766. doi: 10.1002/advs.202406766 (PMC11714174; doi:10.1002/advs.202406766)
Supplement: Supplementary file 1 — Supporting Information [file ADVS-12-2406766-s003.pdf]

ADVANCED SCIENCE
Open Access

Supporting Information

for *Adv. Sci.*, DOI 10.1002/advs.202406766

A Neural Circuit For Bergamot Essential Oil-Induced Anxiolytic Effects

Meng-Yu Zhu, Wan-Ying Dong, Jin-Rong Guo, Ji-Ye Huang, Ping-Kai Cheng, Yumeng Yang, An Liu, Xin-Lu Yang, Xia Zhu, Zhi Zhang, Yuanyin Wang* and Wenjuan Tao**

Supporting Information

A neural circuit for bergamot essential oil-induced anxiolytic effects

Authors: Meng-Yu Zhu[1,2,5], Wan-Ying Dong[3,5], Jin-Rong Guo[2,5], Ji-Ye Huang[3], Ping-Kai Cheng[3], Yumeng Yang[1,2], An Liu[1,2], Xinlu Yang[3], Xia Zhu[3], Zhi Zhang[2,3,4]*, Yuanyin Wang[1]*, Wenjuan Tao[1,2,6]*

Supplemental figure titles and legends

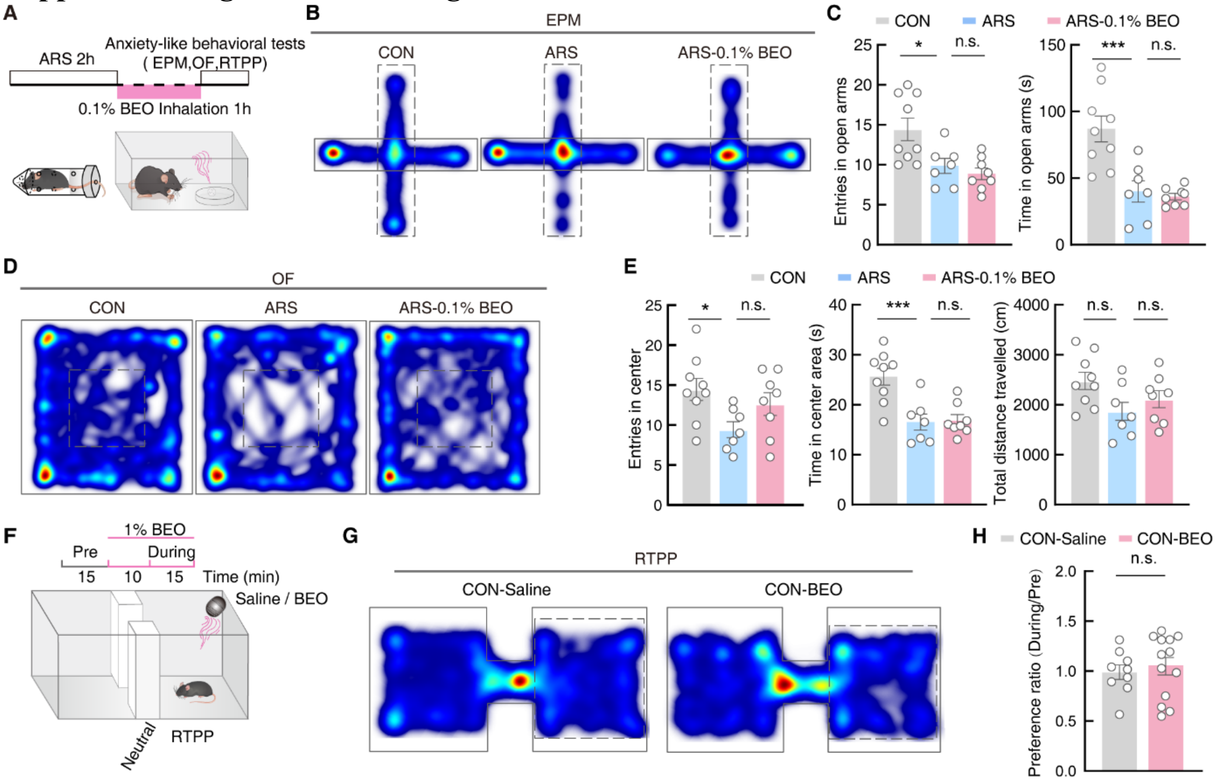

Fig S1. Behavioral effects of 0.1% BEO inhalation at 2h post-ARS in mice.

(A) Experimental paradigm for establishing the acute restraint stress (ARS) mouse model and 0.1% Bergamot Essential Oil (BEO) inhalation. Stress was induced by enclosing mice for 2h in a 50ml plastic syringe with holes drilled for ventilation. Mice were treated with inhalation exposure to 0.1% BEO (ARS-0.1% BEO) or saline control (ARS) for 1h following restraint.

(B-C) Heatmaps of locomotion traces from elevated plus maze (EPM) tests (B) and summarized data of the entries in open arms (C, left) and time in open arms (C, right) in EPM tests (CON, n = 9 mice; ARS, n = 7 mice; ARS-0.1% BEO, n = 8 mice; left, $F_{(2, 21)} = 7.601$, $P = 0.0033$; right, $F_{(2, 21)} = 14.58$, $P = 0.0001$).

(D-E) Heatmaps of locomotion traces from open field (OF) tests (D) and summarized data of entries in center area (E, left), time in center area (E, middle), and total distance travelled (E, right) in OF tests (CON, n = 9 mice; ARS, n = 7 mice; ARS-0.1% BEO, n = 8 mice; left, $F_{(2, 21)}$ =3.592 , $P = 0.0455$; middle, $F_{(2, 21)} = 12.35$, $P = 0.0003$; right, F (2, 21) = 2.400, $P = 0.1151$).

(F) Schematic for Real-time Place Preference (RTPP) tests.

(G-H) Heatmaps of mouse location in RTPP tests and summarized data showing the ratio of time spent in the chamber with BEO or saline before versus during the experimental observation period (CON-Saline, n = 9 mice; CON-BEO, n = 13 mice; $t_{20} = 0.4967$, $P = 0.625$).

All data are presented as means ± SEM. *$P < 0.05$, ***$P < 0.001$; n.s., not significant. For detailed statistical information, see also Table S1.

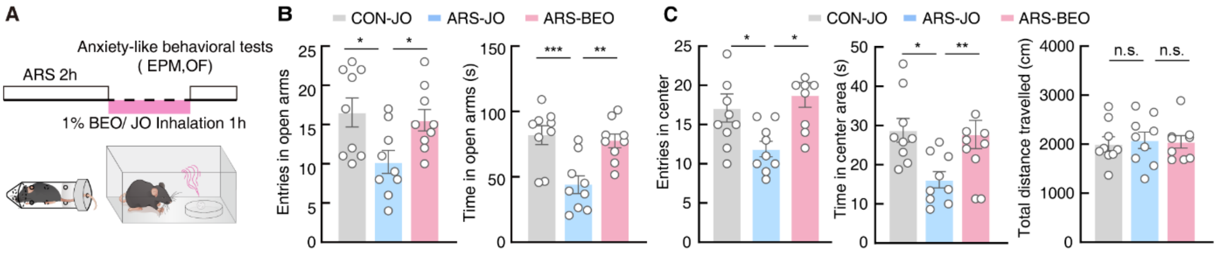

Fig S2. Effects of JO on anxiety-like behaviors in ARS mice.

(A) Experimental paradigm for establishing the ARS mouse model and BEO or JO inhalation. Stress was induced by enclosing mice for 2h in a 50ml plastic syringe with holes drilled for ventilation. Mice were treated with inhalation exposure to BEO (ARS-BEO) or jojoba oil (ARS-JO) for 1h following restraint.

(B) Summarized data of the entries in open arms (left) and time in open arms (right) in EPM tests (CON-JO, n = 9 mice; ARS-JO, n = 9 mice; ARS-BEO, n = 9 mice; left, $F_{(2, 24)} = 4.672$, $P = 0.0193$; right, $F_{(2, 24)} = 10.15$, $P = 0.0006$).

(C) Summarized data of entries in center area (left), time in center area (middle), and total distance travelled (right) in OF tests (CON-JO, n = 9 mice; ARS-JO, n = 9 mice; ARS-BEO, n = 9 mice; left, $F_{(2, 24)} = 5.791$, $P = 0.0089$; middle, $F_{(2, 24)} = 5.574$, $P = 0.0103$; right, $F_{(2, 24)} = 0.06049$, $P = 0.9414$).

All data are presented as means ± SEM. $^{*}P < 0.05$, $^{**}P < 0.01$, $^{***}P < 0.001$; n.s., not significant. For detailed statistical information, see also Table S1.

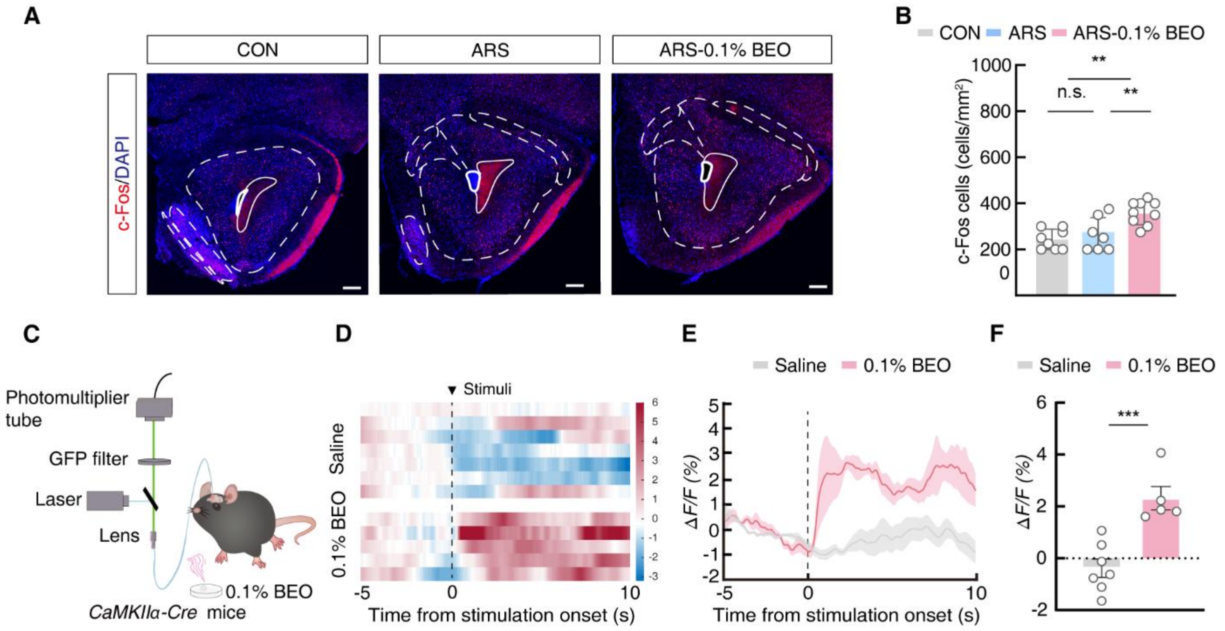

Fig S3. Effects of 0.1% BEO mediates anxiolytic effects through AON$^{CaMKII\alpha}$ neurons in ARS mice.

(A) Representative images of c-Fos^{+} neuron distribution in the AON of ARS, ARS-0.1% BEO, and no stress (controls) mice. Scale bars, 100 μm.

(B) Statistical analysis of c-Fos^{+} neuron distribution in AON between groups (CON, n = 8 mice; ARS, n = 7 mice; ARS-0.1% BEO, n= 9 mice; $F_{(2, 21)} = 10.18$, $P = 0.0008$).

(C) Schematic for *in vivo* fiber photometry recordings.

(D) Heatmaps across mice aligned to the time from onset of 0.1% BEO.

(E-F) Representative traces (E) and averaged *ΔF/F* (F) of AON^{Glu} signals in Saline and 0.1% BEO-treated mice, respectively. Bold lines and light shadow indicate mean and SEM, respectively.

All data are presented as mean ± SEM. $^{**}P < 0.01$, $^{***}P < 0.001$; n.s., not significant. For detailed statistical information, see also Table S1.

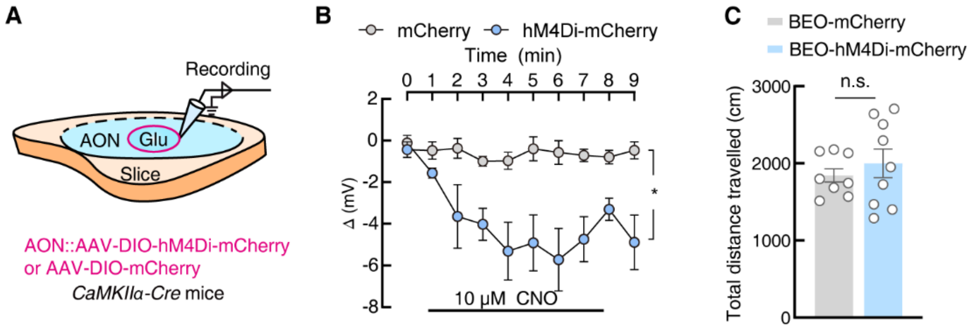

Fig S4. Verification of hM4Di-virus.

(A) Experimental scheme for CNO perfusion and patch clamp recording in AON slices from AAV-DIO-hM4Di-mCherry or AAV-DIO-mCherry-infected *CaMKIIα-Cre* mice.

(B) Whole-cell recordings in acute sections showing the effect of CNO on hM4Di-mCherry or mCherry expressing AON^{Glu} neurons (n = 5 mice per group).

(C) Total distance travelled by BEO-mCherry or BEO-hM4Di-mCherry mice in OF tests (BEO-mCherry, n = 9 mice; BEO-hM4Di-mCherry, n = 8 mice; right, $t_{15} = 0.532$, $P = 0.603$).

All data are presented as means ± SEM. *$P < 0.05$; n.s., not significant. For detailed statistical information, see also Table S1.

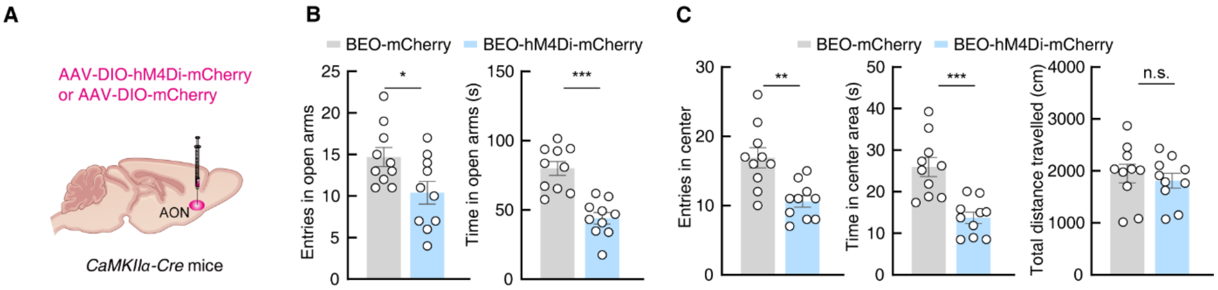

Fig S5. Effects of chemogenetic inhibition of AON$^{CaMKII\alpha}$ neurons in anxiety-like behaviors.

(A) Schematic for AAV-DIO-hM4Di-mCherry or AAV-DIO-mCherry virus injection into the AON of *CaMKIIα-Cre* mice.

(B) Statistical summary of entries in open arms (left) and time in open arms (right) for BEO-mCherry and BEO-hM4Di-mCherry mice in EPM tests with 1 mg/kg CNO injection (BEO-mCherry, n = 10 mice; BEO-hM4Di-mCherry, n = 10 mice; left, $t_{18} = 2.410$, $P = 0.0269$; right, $t_{18} = 5.526$, $P < 0.0001$).

(C) Statistical summary of entries in center (left), time in center areas (middle), and total distance travelled (right) by BEO-mCherry or BEO-hM4Di-mCherry mice in OF tests with 1 mg/kg CNO injection (BEO-mCherry, n = 10 mice; BEO-hM4Di-mCherry, n = 10 mice; left, $t_{18} = 3.726$, $P = 0.0015$; middle, $t_{18} = 4.582$, $P = 0.0002$; right, $t_{15} = 0.1943$, $P = 0.8481$).

All data are presented as means ± SEM. *$P < 0.05$, **$P < 0.01$, ***$P < 0.001$; n.s., not significant. For detailed statistical information, see also Table S1.

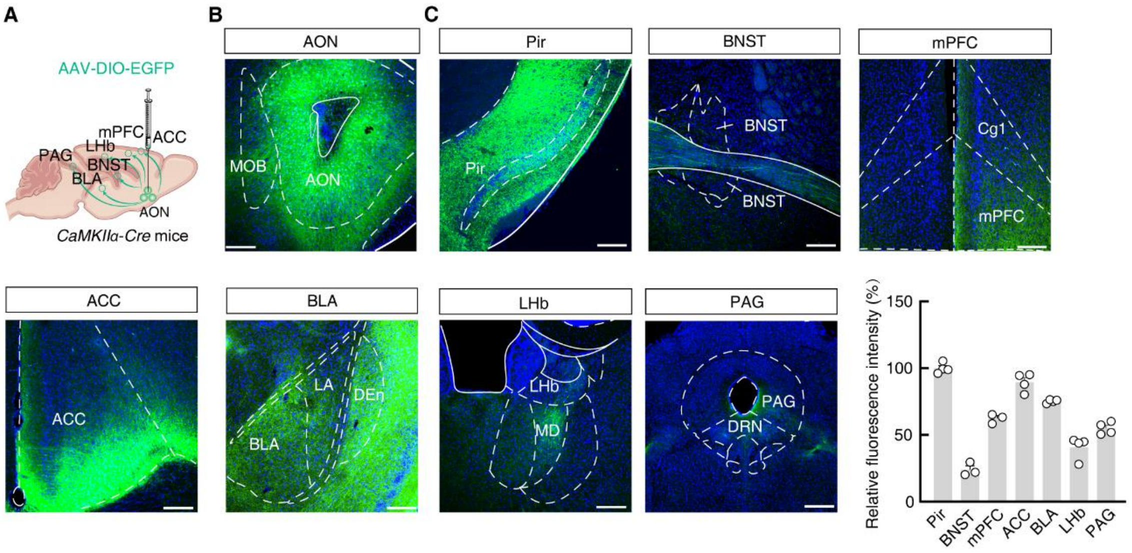

Fig S6. Outputs of AON$^{CaMKII\alpha}$ neurons in *CaMKIIα-Cre* mice..

(A) Schematic of AAV-DIO-EGFP virus injection in the AON of *CaMKIIα-Cre* mice.

(B) Representative image showing AON injection site.

(C) Representative images showing EGFP^{+} fibers in multiple brain regions and summarized data showing the relative fluorescence intensity of EGFP^{+} fibers in the piriform cortex (Pir), bed nucleus of the stria terminalis (BNST), medial prefrontal cortex (mPFC), ACC, basal lateral amygdala (BLA), lateral habenula (LHb), periaqueductal grey (PAG) respectively. Scale bars, 200 μm.

All data are presented as means ± SEM.

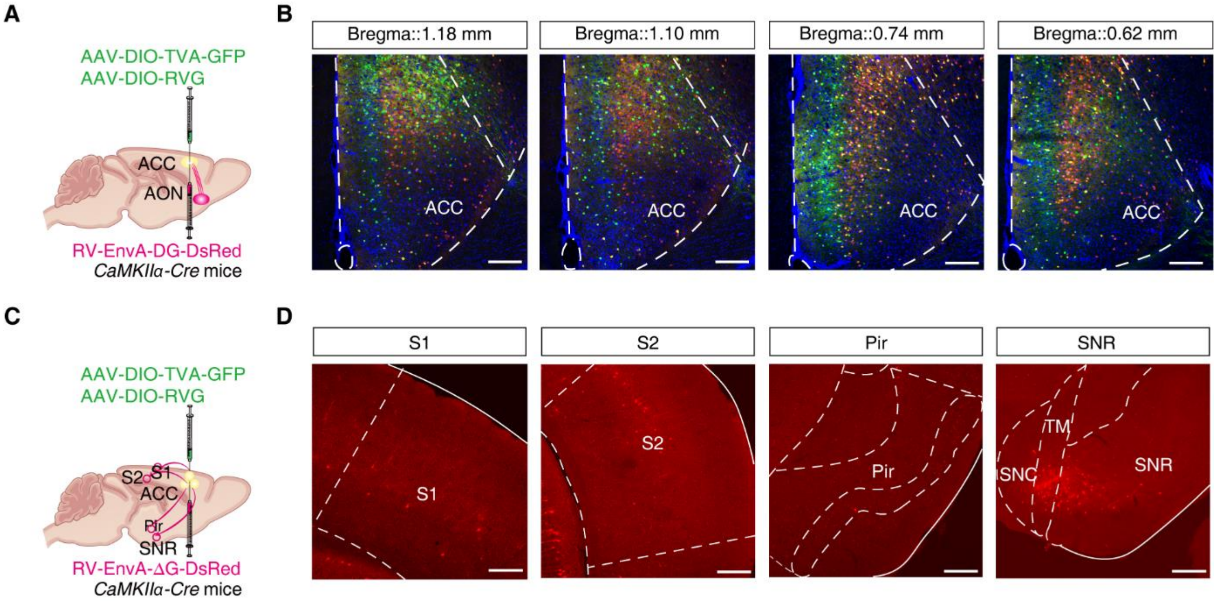

Fig S7. Inputs of ACC neurons of RV tracing strategy of *CaMKIIα-Cre* mice.

(A) Schematic for Cre-dependent retrograde trans-monosynaptic RV tracing strategy in *CaMKIIα-Cre* mice.

(B) The rostro-caudal distribution of DsRed-labelled neurons in the ACC. Scale bars, 100 μm.

(C) Schematic for Cre-dependent retrograde trans-monosynaptic RV tracing strategy in *CaMKIIα-Cre* mice.

(D) The rostro-caudal distribution of DsRed-labelled neurons in multiple brain regions, including the primary somatosensory cortex (S1), secondary somatosensory cortex(S2), the piriform cortex (Pir), and substantia nigra pars reticulata (SNR). Scale bars, 200 μm.

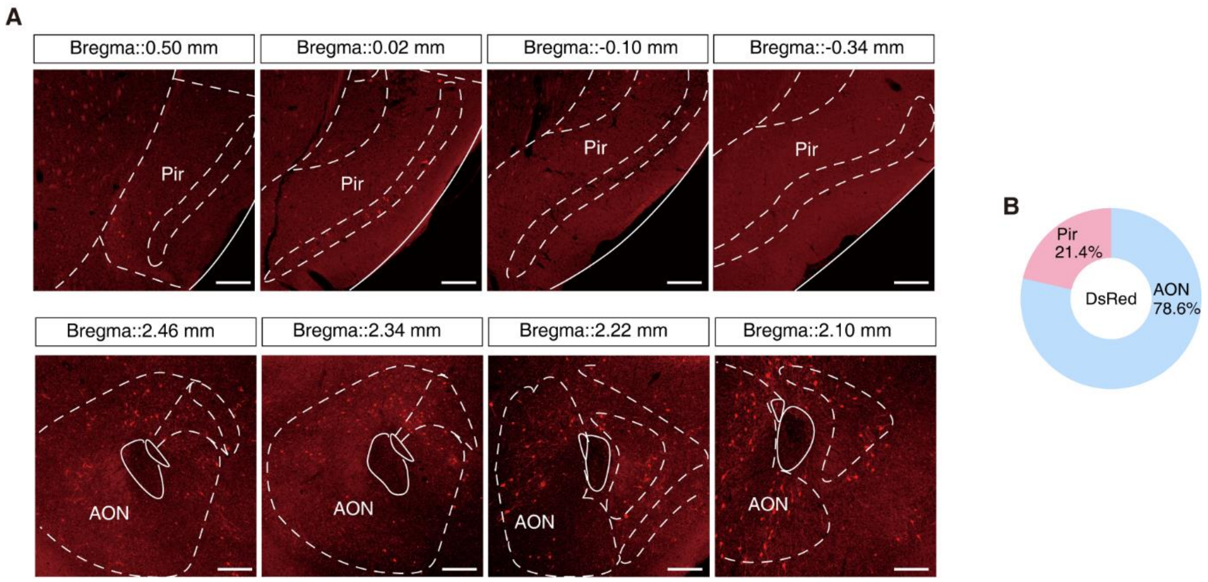

Fig S8. AON neurons input to ACC$^{CaMKII\alpha}$ neurons.

(A) The rostro-caudal distribution of DsRed-labelled neurons in the Pir and AON and Scale bars, 200 μm.

(B) The distribution of DsRed-labeled neurons between AON and Pir in olfactory-related nuclei (n =5 mice per group).

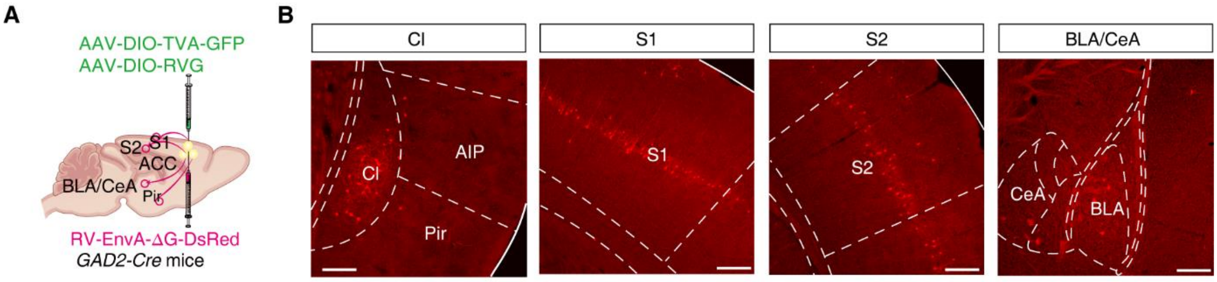

Fig S9. Inputs of ACC neurons of RV tracing strategy of *GAD2-Cre* mice.

(A) Schematic for Cre-dependent retrograde trans-monosynaptic RV tracing strategy in *GAD2-Cre* mice.

(B) The rostro-caudal distribution of DsRed-labelled neurons in multiple brain regions including the claustrum (CI), S1, S2, basal lateral amygdala (BLA) and central amygdala (CeA). Scale bars, 200µm.

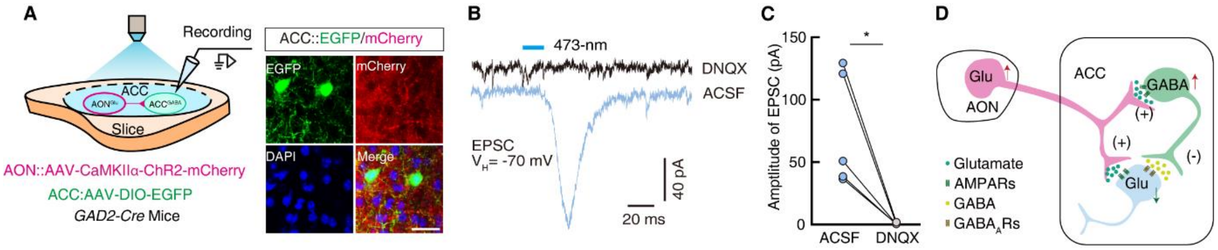

Fig S10. ACC^{GABA} neurons receive AON^{Glu} inputs.

(A) Schematic for the optogenetic activation of AON^{Glu} neuronal terminals with simultaneous whole-cell patch-clamp recordings of EGFP-labeled ACC^{GABA} neurons (left), and representative images of EGFP signals counterstained with GABAergic antibody in the ACC of *GAD2-Cre* mice (right). Scale bars, 20 μm.

(B-C) Representative traces (B) and summarized data (C) from recordings of ACC^{GABA} neurons upon photostimulation of ChR2-expressing AON^{Glu} fibers in slices containing the ACC (EPSC, n = 4 cells, t_3 = 5.596, P = 0.0113).

(D) Illustration of AON^{Glu} projections to ACC neurons.

All data are presented as means ± SEM. *P < 0.05. For detailed statistical information, see also Table S1.

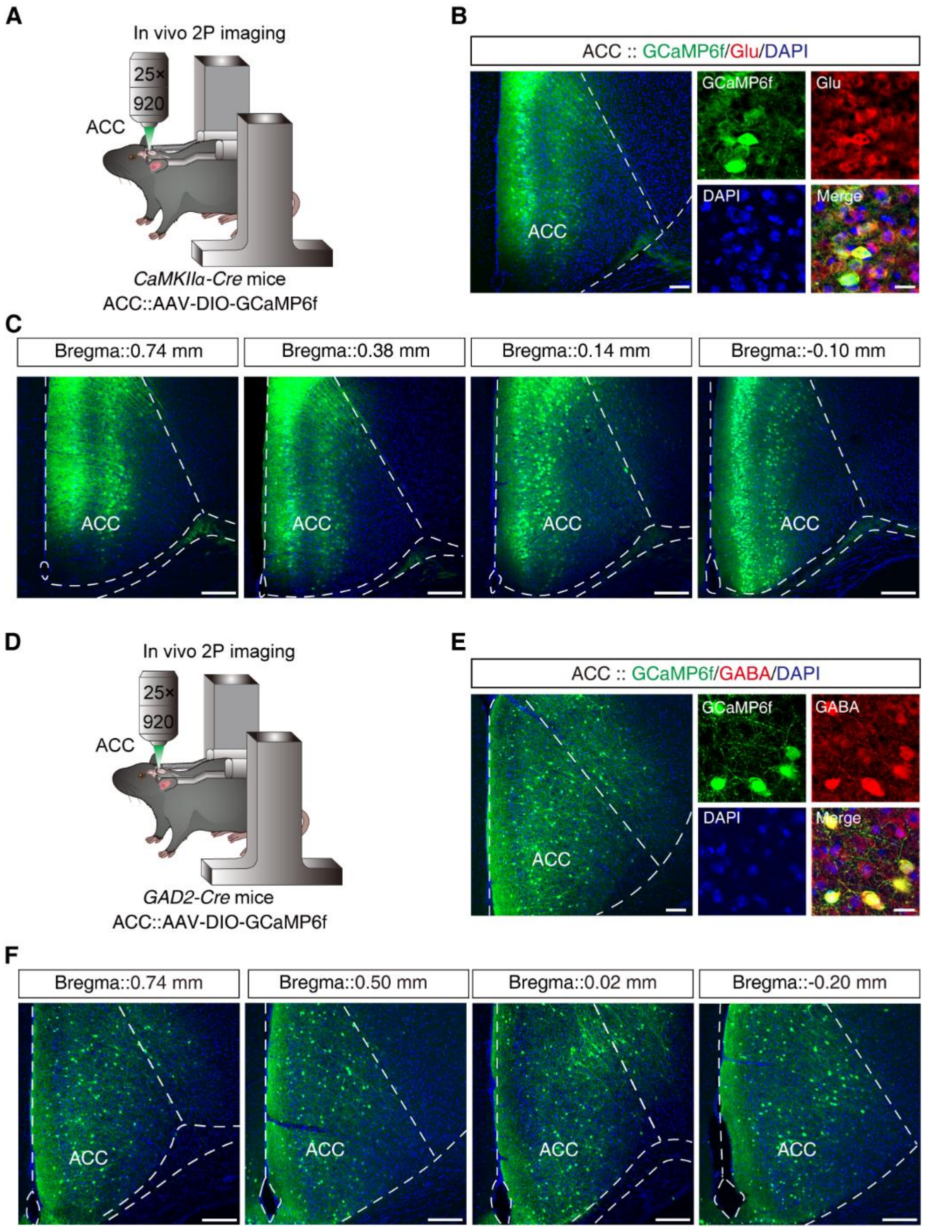

Fig S11. Representative images of GCaMP6f expression in ACC.

(A) Schematic for *in vivo* 2P calcium imaging of ACC$^{CaMKII\alpha}$ neurons in head-restrained *CaMKIIα-Cre* mice.

(B) Representative images of GCaMP6f neurons co-localized with glutamatergic antibody in the ACC. Scale bars, 100 μm (left) and 20 μm (right).

(C) Representative images of GCaMP6f signals in ACC$^{CaMKII\alpha}$ neurons at different bregma sites. Scale bars, 200 μm.

(D) Schematic for *in vivo* 2P calcium imaging of ACC^{GABA} neurons in head-restrained *GAD2-Cre* mice.

(E) Representative images of GCaMP6f neurons co-localized with GABAergic antibody in the ACC. Scale bars, 100 μm (left) and 20 μm (right).

(F) GCaMP6f expression in ACC^{GABA} neurons at different bregma sites. Scale bars, 200 μm.

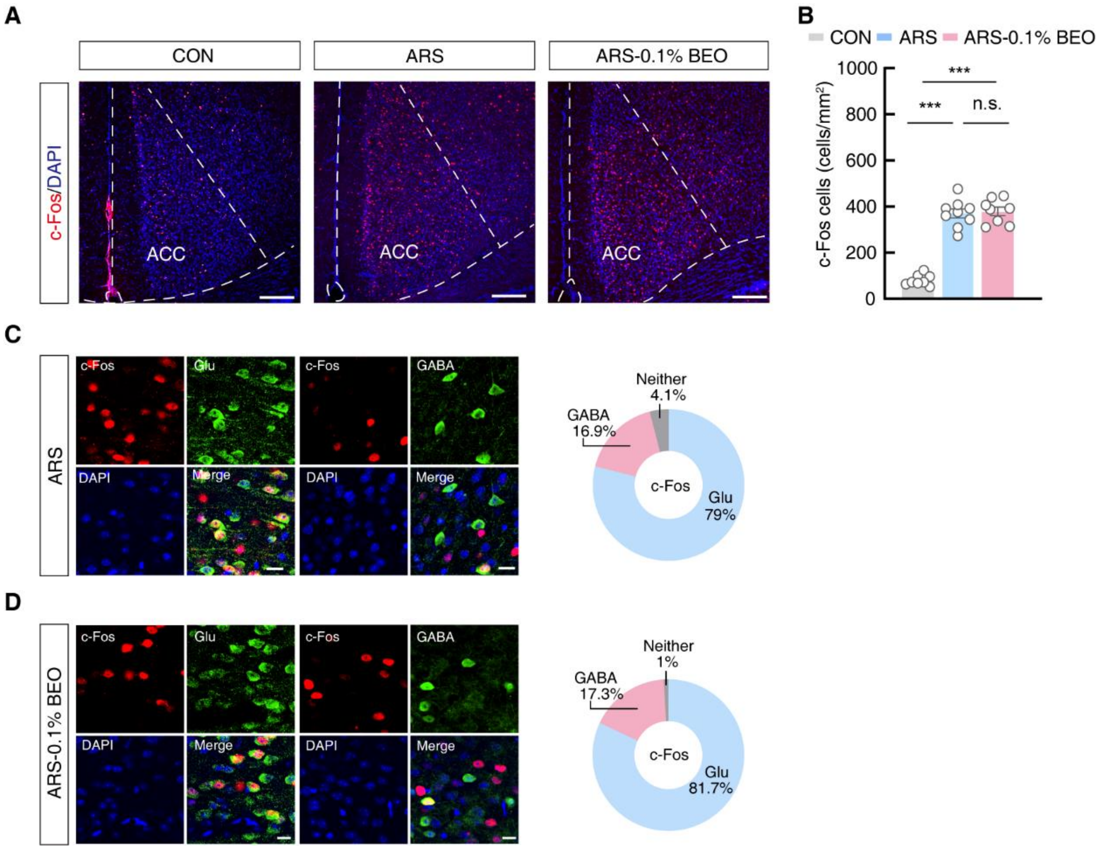

Fig S12. Effects of 0.1% BEO inhalation on ACC activation in ARS mice.

(A) Representative images of c-Fos$^+$ neuron distribution in the ACC of ARS and ARS-0.1% BEO mice. Scale bars, 200 μm.

(B) Statistical analysis of c-Fos$^+$ neuron distribution in the ACC between groups (CON, n = 8 mice; ARS, n = 9 mice; ARS-0.1% BEO, n= 8 mice; $F_{(2, 22)} = 99.69$, $P < 0.0001$).

(C) c-Fos$^+$ neurons co-localized with glutamatergic or GABAergic antibody in the ACC of ARS mice. Scale bar, 20 μm.

(D) c-Fos$^+$ neurons co-localized with glutamatergic or GABAergic antibody in the ACC 0f ARS-0.1% BEO mice. Scale bar, 20 μm.

All data are presented as means ± SEM. *$P < 0.05$, ***$P < 0.001$; n.s., not significant. For detailed statistical information, see also Table S1.

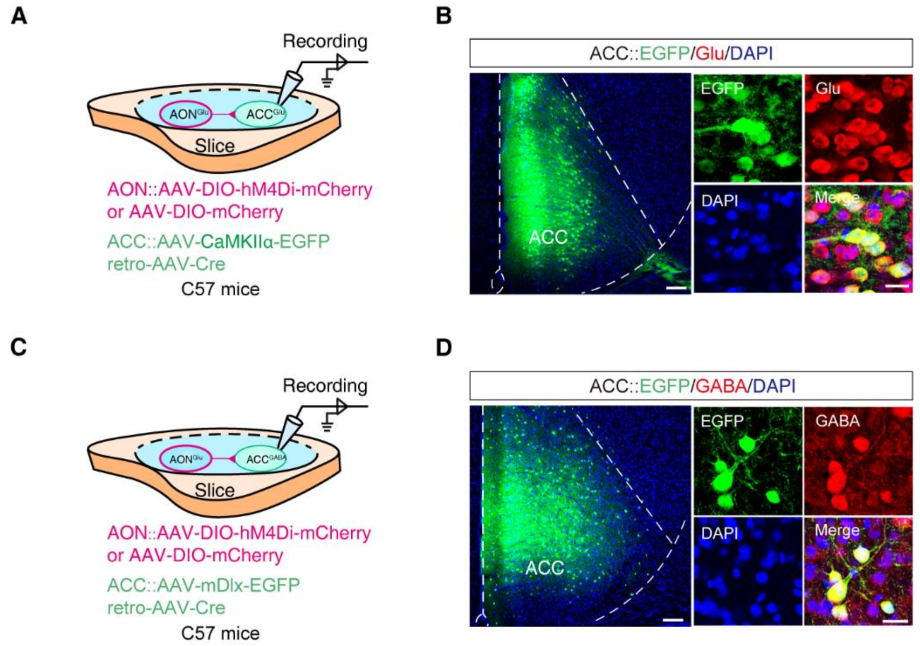

Fig S13. ACC^{Glu} and ACC^{GABA} neuronal activity after inhibition of AON$^{CaMKII\alpha}$.

(A) Schematic of chemogenetic inhibition of ACC-projecting AON$^{CaMKII\alpha}$ neurons and whole-cell patch-clamp recordings of EGFP$^+$ ACC$^{CaMKII\alpha}$ neurons.

(B) EGFP-labeled neurons are co-localized with glutamatergic antibody. Scale bars, 100 μm (left) and 20 μm (right).

(C) Schematic of chemogenetic inhibition of ACC-projecting AON$^{CaMKII\alpha}$ neurons and whole-cell patch-clamp recordings of EGFP$^+$ ACC^{GABA} neurons.

(D) EGFP-labeled neurons are co-localized with GABA neurons. Scale bars, 100 μm (left) and 20 μm (right).

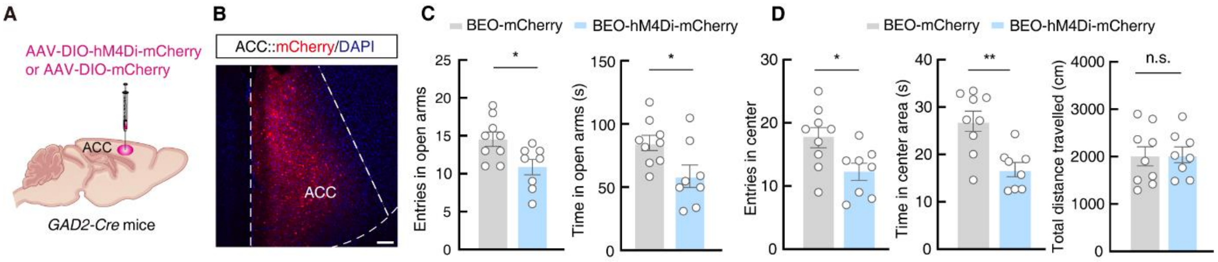

Fig S14. Inhibition of ACC^{GABA} neurons blocked anxiolytic-like effects.

(A) Schematic of AAV-DIO-hM4Di-mCherry or AAV-DIO-mCherry virus injected into the ACC of *GAD2-Cre* mice.

(B) Representative image of the injection site and virus expression in the ACC of *GAD2-Cre* mice.

(C) Summarized data showing the entries in open arms (left) and the time in open arms (right) of BEO-mCherry mice and BEO-hM4Di-mCherry mice (right) with 5 mg/kg CNO injection in EPM tests (BEO-mCherry, n = 9 mice; BEO-hM4Di -mCherry, n = 8 mice; left, t_{15} = 2.666, P = 0.0176; right, t_{15} = 2.494, P = 0.0248).

(D) Summarized data showing the entries in center areas (left), the time in center areas (middle) and total distance travelled (right) of BEO-mCherry mice and BEO-hM4Di-mCherry mice (right) with 5 mg/kg CNO injection in OF tests (BEO-mCherry, n = 9 mice; BEO-hM4Di -mCherry, n = 8 mice; left, t_{15} = 2.537, P = 0.0228; middle, t_{15} = 3.787, P = 0.0018; right, t_{15} = 0.1156, P = 0.9095).

Significance was assessed by two-tailed unpaired Student's t-tests in (C and D). All data are presented as means ± SEM. *P < 0.05, **P < 0.01, n.s., not significant. For detailed statistical information, see also Table S1.

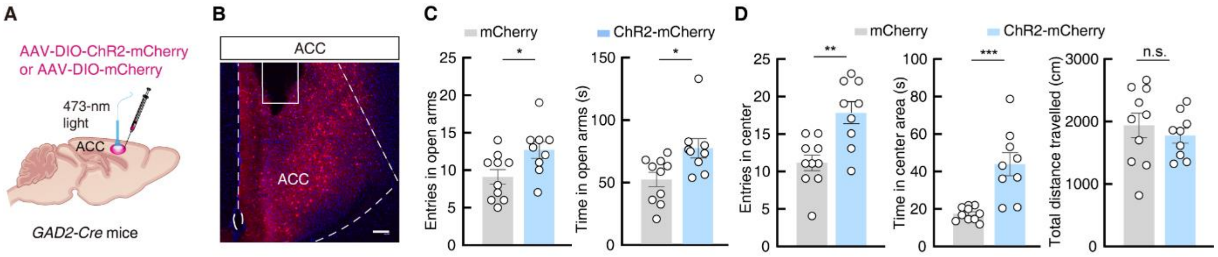

Fig S15. Activation of ACC^{GABA} neurons produces anxiolytic-like effects.

(A) Schematic of AAV-DIO-ChR2-mCherry or AAV-DIO-mCherry virus injected and optical fiber implanted into the ACC of *GAD2*-Cre mice.

(B) Representative image of virus expression and optical fiber implanted in the ACC of *GAD2-Cre* mice. Scale bar, 100 μm.

(C) Summarized data of entries in open arms (left) and time in open arms (right) by mCherry control or ChR2-mCherry mice in EPM tests (mCherry, n = 10 mice; ChR2-mCherry, n = 9 mice; left, $t_{17} = 2.434$, $P = 0.0262$; right, $t_{17} = 2.645$, $P = 0.0170$).

(D) Summarized data showing the entries in center area (left), time in center areas (middle), and total distance travelled (right) by mCherry control and ChR2-mCherry mice in OF tests (mCherry, n = 10 mice; ChR2-mCherry, n = 9 mice; left, $t_{17} = 3.527$, $P = 0.0016$; middle, $t_{17} = 4.396$, $P = 0.0004$; right, $t_{17} = 0.6922$, $P = 0.4981$).

Significance was assessed by two-tailed unpaired Student's t-tests in (C and D). All data are presented as means ± SEM. *$P < 0.05$, **$P < 0.01$, ***$P < 0.001$; n.s., not significant. For detailed statistical information, see also Table S1.

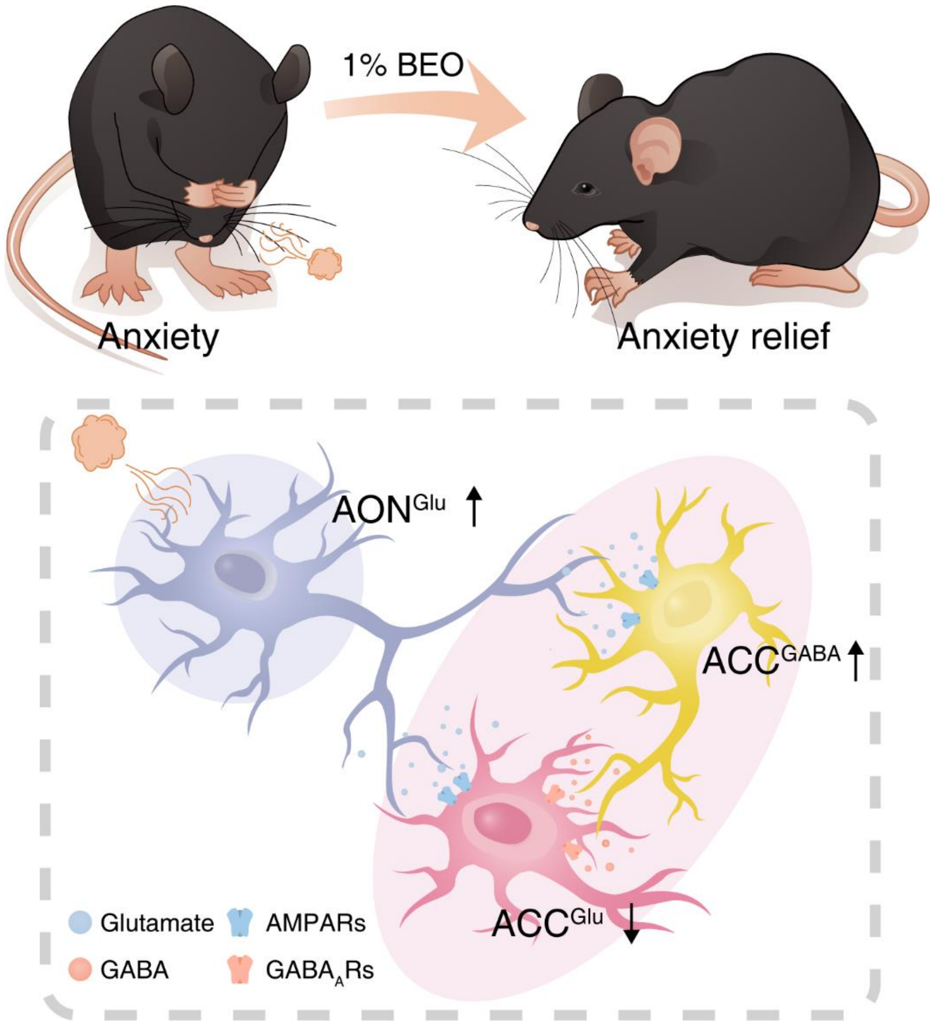

Fig S16. A neural circuit for bergamot essential oil-induced anxiolytic effects.

The circuit involves can activate AON projection to both ACC^{Glu} and ACC^{GABA} neurons. A decrease in the ACC^{Glu} neurons activty is caused by activation of ACC^{GABA} local inteneurons via receiving AON^{Glu} inputs, through which anxiolytic effects is generated.

Video S1. Related to Figure 5

In vivo two-photon imaging for GCaMP6f-expressing ACC^{Glu} neurons in control, ARS and ARS-BEO mice. Scale bar, 100 μm.

Video S2. Related to Figure 5

In vivo two-photon imaging for GCaMP6f-expressing ACC^{GABA} neurons in control, ARS and ARS-BEO mice. Scale bar, 100 μm.

Supplementary Table S1.					
**Extended statistical information for Figures 1-6, and Figures S1-S16.**					
Figure	Conditions (sample size)		Analysis	P value	t/F value
Fig.1C			One-way ANOVA		
Entries in open arms	CON (9)	ARS (7)		*P* = 0.0048	$F_{(2, 20)}$ = 16.67
	ARS (7)	ARS-BEO (7)		*P* = 0.0067	
Time in open arms (s)	CON (9)	ARS (7)		*P* = 0.0052	$F_{(2, 20)}$ = 8.319
	ARS (7)	ARS-BEO (7)		*P* = 0.0065	
Fig.1E			One-way ANOVA		
Entries in center	CON (9)	ARS (7)		*P* = 0.0345	$F_{(2, 20)}$ = 4.607
	ARS (7)	ARS-BEO (7)		*P* = 0.0230	
Time in center area (s)	CON (9)	ARS (7)		*P* = 0.0107	$F_{(2, 20)}$ = 5.979
	ARS (7)	ARS-BEO (7)		*P* = 0.0336	
Total distance travelled (cm)	CON (9)	ARS (7)		*P* = 0.2955	$F_{(2, 20)}$ = 1.473
	ARS (7)	ARS-BEO (7)		*P* = 0.3295	
Fig.1H	ARS-Saline (9)	ARS-BEO (8)	Unpaired two sample t-test	*P* = 0.0215	t_{15} = 0.0215
Fig.2B			One-way ANOVA		
c-Fos cells	CON (7)	ARS (6)		*P* = 0.6102	$F_{(2, 17)}$ = 48.95
	ARS (6)	ARS-BEO (7)		*P* < 0.0001	
Fig.2F	Saline (7)	1%BEO (6)	Unpaired two sample t-test	*P* < 0.0001	t_{11} = 6.204
Fig.2I			Unpaired two sample t-test		

Entries in open arms	BEO-mCherry (9)	BEO-hM4Di-mCherry (9)		P = 0.0001	t_{16} = 4.961
Time in open arms (s)	BEO-mCherry (9)	BEO-hM4Di-mCherry (9)		P = 0.0379	t_{16} = 2.263
Fig.2J			Unpaired two sample t-test		
Entries in center	BEO-mCherry (8)	BEO-hM4Di-mCherry (9)		P = 0.0026	t_{15} = 3.611
Time in center area (s)	BEO-mCherry (8)	BEO-hM4Di-mCherry (9)		P = 0.0189	t_{15} = 2.631
Fig.4D			Paired two sample t-test		
EPSC	ACSF (4)	TTX (4)		P = 0.0113	t_3 = 5.596
EPSC	TTX+4-AP (4)	TTX+4-AP+DNQX (4)		P = 0.0301	t_3 = 3.889
Fig.4E			Paired two sample t-test		
EPSC	EPSC (8)			P = 0.0184	t_7 = 3.059
IPSC	IPSC (5)			P = 0.0152	t_4 = 4.071
Fig.4H	IPSC (6)		Paired two sample t-test	P = 0.0234	t_5 = 3.221
Fig.4K			Unpaired two sample t-test		
Glu	mCherry (8)	ChR2-mCherry (5)		P = 0.6994	t_{11} = 5.059
Fig.4N					
GABA	mCherry (6)	ChR2-mCherry (7)		P = 0.1454	t_{11} = 3.478

Fig.5B			One-way ANOVA		
ΔF/F	CON (5)	ARS (5)		*P* < 0.0001	$F_{(2, 309)}$ = 21.44
	ARS (5)	ARS-BEO (5)		*P* < 0.0001	
Fig.5C			One-way ANOVA		
ΔF/F	CON (5)	ARS (5)		*P* < 0.0001	$F_{(2, 296)}$ = 27.21
	ARS (5)	ARS-BEO (5)		*P* = 0.0089	
Fig.5F					
Firing rate (Hz)	CON-mCherry (28)	BEO-mCherry (26)	Two-way RM ANOVA with Bonferroni post hoc analysis	*P* = 0.0619	$F_{(21, 560)}$ = 2.429
	ARS-mCherry (23)	BEO-mCherry (26)		*P* < 0.0001	
	BEO-hM4Di-mCherry (28)	BEO-mCherry (26)		*P* < 0.0001	
Rehobase (pA)	CON-mCherry (28)	BEO-mCherry (26)	One-way ANOVA	*P* = 0.8060	$F_{(3, 101)}$ = 6.948
	ARS-mCherry (23)	BEO-mCherry (26)		*P* = 0.0006	
	BEO-hM4Di-mCherry (28)	BEO-mCherry (26)		*P* = 0.0073	
Fig.5I					
Firing rate (Hz)	CON-mCherry (22)	BEO-mCherry (21)	Two-way RM ANOVA with Bonferroni post hoc analysis	*P* = 0.0272	$F_{(3, 656)}$ = 43.12

	ARS-mCherry (22)	BEO-mCherry (21)		$P < 0.0001$	
	BEO-hM4Di-mCherry (22)	BEO-mCherry (21)		$P < 0.0001$	
Rehobase (pA)	CON-mCherry (22)	BEO-mCherry (21)	One-way ANOVA	$P = 0.9949$	$F_{(3, 83)} = 4.371$
	ARS-mCherry (22)	BEO-mCherry (21)		$P = 0.0329$	
	BEO-hM4Di-mCherry (22)	BEO-mCherry (21)		$P = 0.0218$	
Fig.6B			Unpaired two sample t-test		
Entries in open arms	mCherry (9)	ChR2- mCherry (9)		$P = 0.0011$	$t_{16} = 3.954$
Time in open arms (s)	mCherry (9)	ChR2- mCherry (9)		$P = 0.0141$	$t_{16} = 2.753$
Fig.6C			Unpaired two sample t-test		
Entries in center	mCherry (9)	ChR2- mCherry (9)		$P = 0.0353$	$t_{16} = 2.299$
Time in center area (s)	mCherry (9)	ChR2- mCherry (9)		$P = 0.0025$	$t_{16} = 3.590$
Total distance travelled (cm)	mCherry (7)	ChR2- mCherry (9)		$P = 0.2869$	$t_{16} = 1.102$
Fig.6E			Unpaired two sample t-test		
Entries in open arms	BEO-mCherry (9)	BEO-hM4Di-mCherry (9)		$P = 0.0007$	$t_{16} = 4.180$
Time in open arms (s)	BEO-mCherry (9)	BEO-hM4Di-mCherry (9)		$P = 0.0029$	$t_{16} = 3.504$

Fig.6F			Unpaired two sample t-test		
Entries in center	BEO-mCherry (9)	BEO-hM4Di-mCherry (9)		*P* = 0.0001	t_{16} = 4.960
Time in center area (s)	BEO-mCherry (9)	BEO-hM4Di-mCherry (9)		*P* = 0.0048	t_{16} = 3.275
Total distance travelled (cm)	BEO-mCherry (9)	BEO-hM4Di-mCherry (9)		*P* = 0.0992	t_{16} = 1.750
Fig.S1C			One-way ANOVA		
Entries in open arms	CON (9)	ARS (7)		*P* = 0.0227	$F_{(2, 21)}$ = 7.601
	ARS (7)	ARS-0.1%BEO (8)		*P* = 0.8200	
Time in open arms (s)	CON (9)	ARS (7)		*P* = 0.0009	$F_{(2, 21)}$ = 14.58
	ARS (7)	ARS-0.1%BEO (8)		*P* = 0.9350	
Fig.S1E			One-way ANOVA		
Entries in center	CON (9)	ARS (7)		*P* = 0.0262	$F_{(2, 21)}$ = 3.592
	ARS (7)	ARS -0.1%BEO (8)		*P* = 0.1904	
Time in center area (s)	CON (9)	ARS (7)		*P* = 0.0007	$F_{(2, 21)}$ = 12.35
	ARS (7)	ARS-0.1%BEO (8)		*P* = 0.9646	
Total distance travelled (cm)	CON (9)	ARS (7)		*P* = 0.1160	$F_{(2, 21)}$ = 2.400
	ARS (7)	ARS-0.1%BEO (8)		*P* = 0.8233	
Fig.S1H	CON-Saline (9)	CON-BEO (13)	Unpaired two sample t-test	*P* = 0.6248	t_{20} = 0.4967

Fig.S2B			One-way ANOVA		
Entries in open arms	CON-JO (9)	ARS-JO (9)		P = 0.0168	$F_{(2, 24)}$ = 4.672
	ARS-JO (9)	ARS-BEO (9)		P = 0.0455	
Time in open arms (s)	CON-JO (9)	ARS-JO (9)		P = 0.0008	$F_{(2, 24)}$ = 10.15
	ARS-JO (9)	ARS-BEO (9)		P = 0.0025	
Fig.S2C			One-way ANOVA		
Entries in center	CON-JO (9)	ARS-JO (9)		P = 0.008	$F_{(2, 24)}$ = 5.791
	ARS-JO (9)	ARS-BEO (9)		P = 0.0235	
Time in center area (s)	CON-JO (9)	ARS-JO (9)		P = 0.0114	$F_{(2, 24)}$ = 5.574
	ARS-JO (9)	ARS-BEO (9)		P = 0.0205	
Total distance travelled (cm)	CON-JO (9)	ARS-JO (9)		P = 0.9161	$F_{(2, 24)}$ = 0.06049
	ARS-JO (9)	ARS-BEO (9)		P = 0.9803	
Fig.S3B			One-way ANOVA		
c-Fos cells	CON (8)	ARS (7)		P = 0.7023	$F_{(2, 21)}$ = 10.18
	ARS (7)	ARS-0.1%BEO (9)		P = 0.0058	
Fig.S3F	Saline (7)	0.1%BEO (5)	Unpaired two sample t-test	P = 0.0008	t_{10}= 4.775
Fig.S4C	BEO-mCherry (8)	BEO-hM4Di-mCherry (9)	Unpaired two sample t-test	P = 0.6028	t_{15} = 0.5315
Fig.S5B			Unpaired two sample t-test		
Entries in open arms	BEO-mCherry (10)	BEO-hM4Di-mCherry (10)		P = 0.0269	t_{18} = 2.410

Time in open arms (s)	BEO-mCherry (10)	BEO-hM4Di-mCherry (10)		*P* < 0.0001	t_{18} = 5.526
Fig.S5C			Unpaired two sample t-test		
Entries in center	BEO-mCherry (10)	BEO-hM4Di-mCherry (10)		*P* = 0.0015	t_{18} = 3.726
Time in center area (s)	BEO-mCherry (10)	BEO-hM4Di-mCherry (10)		*P* = 0.0002	t_{18} = 4.582
Total distance travelled (cm)	BEO-mCherry (10)	BEO-hM4Di-mCherry (10)		*P* = 0.8481	t_{18} = 0.1943
Fig.S10	ACSF (5)	DNQX (5)	Paired two sample t-test	*P* = 0.0113	t_3 = 5.596
Fig.S12B			One-way ANOVA		
c-Fos cells	CON (8)	ARS (9)		*P* < 0.0001	$F_{(2, 22)}$ = 99.69
	ARS (9)	ARS-0.1%BEO (8)		*P* = 0.8915	
Fig.S14C			Unpaired two sample t-test		
Entries in open arms	BEO-mCherry (9)	BEO-hM4Di-mCherry (8)		*P* = 0.0176	t_{15} = 2.666
Time in open arms (s)	BEO-mCherry (9)	BEO-hM4Di-mCherry (8)		*P* = 0.0248	t_{15} = 2.494
Fig.S14D			Unpaired two sample t-test		
Entries in center	BEO-mCherry (9)	BEO-hM4Di-mCherry (8)		*P* = 0.0228	t_{15} = 2.537

Time in center area (s)	BEO-mCherry (9)	BEO-hM4Di-mCherry (8)		*P* = 0.0018	t_{15} = 3.787
Total distance travelled (cm)	BEO-mCherry (9)	BEO-hM4Di-mCherry (8)		*P* = 0.9095	t_{15} = 0.1156
Fig.S15C			Unpaired two sample t-test		
Entries in open arms	mCherry (10)	ChR2-mCherry (9)		*P* = 0.0262	t_{17} = 2.434
Time in open arms (s)	mCherry (10)	ChR2-mCherry (9)		*P* = 0.0170	t_{17} = 2.645
Fig.S15D			Unpaired two sample t-test		
Entries in center	mCherry (10)	ChR2-mCherry (9)		*P* = 0.0016	t_{17} = 3.527
Time in center area (s)	mCherry (10)	ChR2-mCherry (9)		*P* = 0.0004	t_{17} = 4.396
Total distance travelled (cm)	mCherry (10)	ChR2-mCherry (9)		*P* = 0.4981	t_{17} = 0.6922
